# Supplementary material for: OXPHOS deficiencies affect peroxisome proliferation by downregulating genes controlled by the SNF1 signaling pathway
Source: eLife. 2022 Apr 25;11:e75143. doi: 10.7554/eLife.75143 (PMC9094750; doi:10.7554/eLife.75143)
Supplement: Supplementary file 1. [file elife-75143-supp1.docx]

| **Name** | **Stock name** | **Background** | **Description** | **Genotype** | **References / Sources** |
| --- | --- | --- | --- | --- | --- |
| WT | GS115 | NRRL Y-11430 | WT | *his4* | [1] |
| WT | PPY12 | NRRL Y-11430 | WT | *his4 arg4* | [2] |
| WT | PPF1 | NRRL Y-11430 | WT | *his4 arg4* | [3] |
| Δ*gal83* | Δ*PAS_chr1-4_0498* | GS115 | Δ*gal83* | GS115 Δ*gal83*::*ZEO* *his4* | [4] |
| Δ*sak1* | Δ*PAS_chr2-1_0639* | GS115 | Δ*sak1* | GS115 Δ*sak1*::*ZEO* *his4* | [4] |
| Δ*tos3?* | Δ*PAS_chr1-3_0213* | GS115 | Δ*tos3?* | GS115 Δ*PAS_chr1-3_0213*::*ZEO* *his4* | [4] |
| Δ*hog1* | Δ*PAS_chr1-3_0232* | GS115 | Δ*hog1* | GS115 Δ*hog1*::*ZEO his4* | [4] |
| Δ*pka_B* | Δ*PAS_chr3_0964* | GS115 | Δ*pka_B* | GS115 Δ*PAS_chr3_0964*::*ZEO his4* | [4] |
| Δ*mig1* Δ*mig2* Δ*nrg1* | Δ*mig1* Δ*mig2* Δ*nrg1* | GS115 | Δ*mig1* Δ*mig2* Δ*nrg1* | GS115 Δ*mig1*::*ZEO* Δ*mig2*::*KAN* Δ*nrg1*::*HPH* *his4* | [5] |
| Δ*mxr1* | JC132 | GS115 | *mxr1-1* | GS115 *mxr1-1 his4* | [6] |
| Δ*pex14* | JC404 | NRRL Y-11430 | Δ*pex14* | NRRL Y-11430 Δ*pex14*::*ARG4* *his4* | [7] |
| Δ*aox1* Δ*aox2* | MC100-3 | PPF1 | Δ*aox1* Δ*aox2* | PPF1 Δ*aox1*::*ARG4* Δ*aox2*::*HIS4* | [8] |
| Δ*pex5* | *pas8* | PPY12 | Δ*pex5* | PPY12 Δ*pex5*::*ARG4* *his4* | [9] |
| Δ*pot1* | Δ*pot1* | PPY12 | Δ*pot1* | PPY12 Δ*pot1*::*ARG4* *his4* | [10] |
| WT-OE-Mit1 | WT-Mit1 | GS115 | WT + P*_GAPDH_*, Mit1 | GS115 P*_GAPDH_*::pP6GM1(P*_GAPDH_*, Mit1; *HIS4*, *BLA*) | [5] |
| Δ*nugM* | SJCF2769 | GS115 | Δ*nugM* | GS115 Δ*nugM*::*NAT his4* | This study |
| Δ*gpdA* Δ*mdhB* | Δ*gpdA* Δ*mdhB* | GS115 | Δ*gpdA* Δ*mdhB* | GS115 Δ*gpdA*::*NAT* Δ*mdhB*::*HPH his4* | This study |
| Δ*cat2* | Δ*cat2* | GS115 | Δ*cat2* | GS115 Δ*cat2*::*NAT his4* | This study |
| WT + Pex3-GFP + BFP-SKL | SJCF2177 | SEW1 | Δ*pex3* + P*_PEX3_*, Pex3-GFP + P*_GAPDH_*, BFP-SKL | PPY12 Δ*pex3*::*ARG4* *his4*::pJCF533(P*_PEX3_*, Pex3-GFP; *HIS4*) P*_GAPDH_*::pJCF742(P*_GAPDH_*, BFP-SKL; *ZEO*) | This study |
| Δ*cat2* + Pex3-GFP + BFP-SKL | SJCF2177 | SJCF2177 | Δ*cat2* Δ*pex3* + P*_PEX3_*, Pex3-GFP + P*_GAPDH_*, BFP-SKL | PPY12 Δ*cat2*::*NAT* Δ*pex3*::*ARG4* *his4*::pJCF533(P*_PEX3_*, Pex3-GFP; *HIS4*) P*_GAPDH_*::pJCF742(P*_GAPDH_*, BFP-SKL; *ZEO*) | This study |
| Δ*gpdA* + Pex3-GFP + BFP-SKL | SJCF2649 | SJCF2177 | Δ*gpdA* Δ*pex3* + P*_PEX3_*, Pex3-GFP + P*_GAPDH_*, BFP-SKL | PPY12 Δ*gpdA*::*NAT* Δ*pex3*::*ARG4* *his4*::pJCF533(P*_PEX3_*, Pex3-GFP; *HIS4*) P*_GAPDH_*::pJCF742(P*_GAPDH_*, BFP-SKL; *ZEO*) | This study |
| Δ*mdhA* + Pex3-GFP + BFP-SKL | SJCF2650 | SJCF2177 | Δ*mdhA* Δ*pex3* + P*_PEX3_*, Pex3-GFP + P*_GAPDH_*, BFP-SKL | PPY12 Δ*mdhA*::*HPH* Δ*pex3*::*ARG4* *his4*::pJCF533(P*_PEX3_*, Pex3-GFP; *HIS4*) P*_GAPDH_*::pJCF742(P*_GAPDH_*, BFP-SKL; *ZEO*) | This study |
| Δ*mdhB* + Pex3-GFP + BFP-SKL | SJCF2651 | SJCF2177 | Δ*mdhB* Δ*pex3* + P*_PEX3_*, Pex3-GFP + P*_GAPDH_*, BFP-SKL | PPY12 Δ*mdhB*::*HPH* Δ*pex3*::*ARG4* *his4*::pJCF533(P*_PEX3_*, Pex3-GFP; *HIS4*) P*_GAPDH_*::pJCF742(P*_GAPDH_*, BFP-SKL; *ZEO*) | This study |
| Δ*gpdA* Δ*mdhA* + Pex3-GFP + BFP-SKL | SJCF2652 | SJCF2177 | Δ*gpdA* Δ*mdhA* Δ*pex3* + P*_PEX3_*, Pex3-GFP + P*_GAPDH_*, BFP-SKL | PPY12 Δ*gpdA*::*NAT* Δ*mdhA*::*HPH* Δ*pex3*::*ARG4* *his4*::pJCF533(P*_PEX3_*, Pex3-GFP; *HIS4*) P*_GAPDH_*::pJCF742(P*_GAPDH_*, BFP-SKL; *ZEO*) | This study |
| Δ*gpdA* Δ*mdhB* + Pex3-GFP + BFP-SKL | SJCF2653 | SJCF2177 | Δ*gpdA* Δ*mdh*B Δ*pex3* + P*_PEX3_*, Pex3-GFP + P*_GAPDH_*, BFP-SKL | PPY12 Δ*gpdA*::*NAT* Δ*mdhB*::*HPH* Δ*pex3*::*ARG4* *his4*::pJCF533(P*_PEX3_*, Pex3-GFP; *HIS4*) P*_GAPDH_*::pJCF742(P*_GAPDH_*, BFP-SKL; *ZEO*) | This study |
| Δ*ndufa9* + Pex3-GFP + BFP-SKL | SJCF2597 | SJCF2177 | Δ*ndufa9* Δ*pex3* + P*_PEX3_*, Pex3-GFP + P*_GAPDH_*, BFP-SKL | PPY12 Δ*ndufa9*::*NAT* Δ*pex3*::*ARG4* *his4*::pJCF533(P*_PEX3_*, Pex3-GFP; *HIS4*) P*_GAPDH_*::pJCF742(P*_GAPDH_*, BFP-SKL; *ZEO*) | This study |
| Δ*nugM* + Pex3-GFP + BFP-SKL | SJCF2631 | SJCF2177 | Δ*nugM* Δ*pex3* + P*_PEX3_*, Pex3-GFP + P*_GAPDH_*, BFP-SKL | PPY12 Δ*nugM*::*NAT* Δ*pex3*::*ARG4* *his4*::pJCF533(P*_PEX3_*, Pex3-GFP; *HIS4*) P*_GAPDH_*::pJCF742(P*_GAPDH_*, BFP-SKL; *ZEO*) | This study |
| Δ*cyt1* + Pex3-GFP + BFP-SKL | SJCF2645 | SJCF2177 | Δ*cyt1* Δ*pex3* + P*_PEX3_*, Pex3-GFP + P*_GAPDH_*, BFP-SKL | PPY12 Δ*cyt1*::*NAT* Δ*pex3*::*ARG4* *his4*::pJCF533(P*_PEX3_*, Pex3-GFP; *HIS4*) P*_GAPDH_*::pJCF742(P*_GAPDH_*, BFP-SKL; *_ZEO_*) | This study |
| Δ*aox1* Δ*aox2* + Pex3-GFP + BFP-SKL | SJCF2671 | PPF1 | Δ*aox1* Δ*aox2* P*_PEX3_*, Pex3-GFP + P*_GAPDH_*, BFP-SKL | PPF1 Δ*aox1*::*ARG4* Δ*aox2*::*HIS4* *HIS4*::pJCF571(P*_PEX3_*, Pex3-GFP; *HIS4*, *ZEO*) *HIS4*::pJCF401(P*_GAPDH_*, BFP-SKL; *HIS4*, *KAN*) | This study |
| Δ*pot1* + Pex3-GFP + BFP-SKL | SJCF2655 | Δ*pot1* | Δ*pot1* + P*_PEX3_*, Pex3-GFP + P*_GAPDH_*, BFP-SKL | PPY12 Δ*pot1*::*ARG4* *PEX3*::pJCF852(P*_PEX3_*, Pex3-GFP; *HIS4*) P*_GAPDH_*::pJCF742(P*_GAPDH_*, BFP-SKL; *ZEO*) | This study |
| WT + GFP-Pex36 | SJCF1448 | GS115 | WT + P*_PEX36_*, GFP-Pex36 | GS115 *his4*::pJCF205(P*_PEX36_*, GFP-Pex36; *HIS4*) | This study |
| Δ*pex5* + GFP-Pex36 | SJCF683 | PPY12 | Δ*pex5* + P*_PEX36_*, GFP-Pex36 | PPY12 Δ*pex5*::*ARG4* *his4*::pJCF205(P*_PEX36_*, GFP-Pex36; *HIS4*) | This study |
| WT + Pex11-2HA | SJCF1355 | GS115 | WT + P*_PEX11_*, Pex11-2HA | GS115 *his4*::pMY59(P*_PEX11_*, Pex11-2HA; *HIS4*) | This study |
| Δ*nugM* + Pex11-2HA | SJCF2709 | SJCF1355 | Δ*nugM* + P*_PEX11_*, Pex11-2HA | GS115 Δ*nugM*::*NAT his4*::pMY59(P*_PEX11_*, Pex11-2HA; *HIS4*) | This study |
| Δ*gal83* + Pex11-2HA | SJCF2719 | Δ*PAS_chr1-4_0498* | Δ*gal83* + P*_PEX11_*, Pex11-2HA | GS115 Δ*gal83*::*ZEO* *his4*::pMY59(P*_PEX11_*, Pex11-2HA; *HIS4*) | This study |
| Δ*sak1* + Pex11-2HA | SJCF2720 | Δ*PAS_chr2-1_0639* | Δ*sak1* + P*_PEX11_,* Pex11-2HA | GS115 Δ*sak1*::*ZEO* *his4*::pMY59(P*_PEX11_*, Pex11-2HA; *HIS4*) | This study |
| Δ*tos3?* + Pex11-2HA | SJCF2718 | Δ*PAS_chr1-3_0213* | Δ*tos3?* + P*_PEX11_*, Pex11-2HA | GS115 Δ*PAS_chr1-3_0213*::*ZEO* *his4*::pMY59(P*_PEX11_*, Pex11-2HA; *HIS4*) | This study |
| Δ*mxr1* | Δ*mxr1* + pIB1 | JC132 | *mxr1-1* | GS115 *mxr1-1 his4*::pIB1(empty plasmid; *HIS4*) | This study |
| Δ*mit1* | Δ*mit1* + pIB1 + pJCF214 | SMY288 | Δ*mit1* | PPY12 Δ*mit1*::*ZEO* *his4*::pIB1(empty plasmid; *HIS4*) *arg4*::pJCF214(empty plasmid; *ARG4*) | This study |
| Δ*pka_B* + Pex3-GFP | SJCF2724 | Δ*PAS_chr3_0964* | Δ*pka_B* + P*_PEX3_*, Pex3-GFP | GS115 Δ*pka_B*::*ZEO* *PEX3*::pJCF852(P*_PEX3_*, Pex3-GFP; *HIS4*) | This study |
| Δ*pka_B* *pka_A^M219G^* + Pex3-GFP | SJCF2735 | SJCF2724 | Δ*pka_B* *pka_A*^M219G^ + P*_PEX3_*, Pex3-GFP | GS115 *PKA_A*::*pka_A*^M219G^(*KAN*) Δ*PAS_chr3_0964*::*ZEO* *PEX3*::pJCF852(P*_PEX3_*, Pex3-GFP; *HIS4*) | This study |
| Δ*pka_B* *pka_A^M219G^* Δ*nugM* + Pex3-GFP | SJCF2736 | SJCF2735 | Δ*nugM* Δ*pka_B* *pka_A*^M219G^ + P*_PEX3_*, Pex3-GFP | GS115 Δ*nugM*::*NAT* *PKA_A*::*pka_A*^M219G^(*KAN*) Δ*PAS_chr3_0964*::*ZEO* *PEX3*::pJCF852(P*_PEX3_*, Pex3-GFP; *HIS4*) | This study |
| WT + Pex3-GFP | SJCF2722 | GS115 | WT + P*_PEX3_*, Pex3-GFP | GS115 *PEX3*::pJCF852(P*_PEX3_*, Pex3-GFP; *HIS4*) | This study |
| Δ*gal83* + Pex3-GFP | SJCF2727 | Δ*PAS_chr1-4_0498* | Δ*gal83* + P*_PEX3_*, Pex3-GFP | GS115 Δ*gal83*::*ZEO* *PEX3*::pJCF852(P*_PEX3_*, Pex3-GFP; *HIS4*) | This study |
| Δ*hog1* + Pex3-GFP | SJCF2729 | Δ*PAS_chr1-3_0232* | Δ*hog1* + P*_PEX3_*, Pex3-GFP | GS115 Δ*hog1*::*ZEO* *PEX3*::pJCF852(P*_PEX3_*, Pex3-GFP; *HIS4*) | This study |
| Δ*nugM* + Pex3-GFP | SJCF2730 | SJCF2722 | Δ*nugM* + P*_PEX3_*, Pex3-GFP | GS115 Δ*nugM*::*NAT* *PEX3*::pJCF852(P*_PEX3_*, Pex3-GFP; *HIS4*) | This study |
| Δ*hog1* Δ*nugM* + Pex3-GFP | SJCF2734 | SJCF2729 | Δ*nugM* Δ*hog1* + P*_PEX_*_3_, Pex3-GFP | GS115 Δ*nugM*::*NAT* Δ*hog1*::*ZEO* *PEX3*::pJCF852(P*_PEX3_*, Pex3-GFP; *HIS4*) | This study |
| Δ*mig1* Δ*mig2* Δ*nrg1* + Pex3-GFP | SJCF2777 | Δ*mig1* Δ*mig2* Δ*nrg1* | Δ*mig1* Δ*mig2* Δ*nrg1* + P*_PEX3_*, Pex3-GFP | GS115 Δ*mig1*::*ZEO* Δ*mig2*::*KAN* Δ*nrg1*::*HPH* *PEX3*::pJCF852(P*_PEX3_*, Pex3-GFP; *HIS4*) | This study |
| Δ*mig1* Δ*mig2* Δ*nrg1* Δ*gal83* + Pex3-GFP | SJCF2778 | SJCF2777 | Δ*mig1* Δ*mig2* Δ*nrg1* Δ*gal83* + P*_PEX3_*, Pex3-GFP | GS115 Δ*gal83*::*NAT* Δ*mig1*::*ZEO* Δ*mig2*::*KAN* Δ*nrg1*::*HPH* *PEX3*::pJCF852(P*_PEX3_*, Pex3-GFP; *HIS4*) | This study |
| Δ*mig1* Δ*mig2* Δ*nrg1* Δ*nugM* + Pex3-GFP | SJCF2779 | SJCF2777 | Δ*mig1* Δ*mig2* Δ*nrg1* Δ*nugM* + P*_PEX3_*, Pex3-GFP | GS115 Δ*nugM*::*NAT* Δ*mig1*::*ZEO* Δ*mig2*::*KAN* Δ*nrg1*::*HPH* *PEX3*::pJCF852(P*_PEX3_*, Pex3-GFP; *HIS4*) | This study |
| WT + Pex3-GFP | SJCF1520 | GS115 | WT + P*_PEX3_*, Pex3-GFP | GS115 *his4*::pJCF533(P*_PEX3_*, Pex3-GFP; *HIS4*) | This study |
| Δ*pex14* + Pex3-GFP | SJCF2672 | JC404 | Δ*pex14* + P*_PEX3_*, Pex3-GFP | NRRL Y-11430 Δ*pex14*::*ARG4* *his4*::pJCF533(P*_PEX3_*, Pex3-GFP; *HIS4*) | This study |
| Δ*pex11* + Pex3-GFP | SJCF1565 | SJCF1555 | Δ*pex11* + P*_PEX3_*, Pex3-GFP | GS115 Δ*pex11*::*ZEO* *his4*::pJCF533(P*_PEX3_*, Pex3-GFP; *HIS4*) | This study |
| WT + Gal83-GFP | SJCF2772 | GS115 | WT + P*_GAL83_*, Gal83-GFP | GS115 *his4*::P*_GAL83_*, Gal83-GFP(*HIS4*) | This study |
| Δ*nugM* + Gal83-GFP | SJCF2773 | SJCF2772 | Δ*nugM* + P*_GAL83_*, Gal83-GFP | GS115 Δ*nugM*::*NAT* *his4*::P*_GAL83_*, Gal83-GFP(*HIS4*) | This study |
| Δ*sak1* + Gal83-GFP | SJCF2774 | Δ*PAS_chr2-1_0639* | Δ*sak1* + P*_GAL83_*, Gal83-GFP | GS115 Δsak1::*ZEO his4*::P*_GAL83_*, Gal83-GFP(*HIS4*) | This study |
| WT + Gal83-GFP + Sec61-mCherry | SJCF2775 | SJCF2772 | WT + P*_GAL83_*, Gal83-GFP + P*_SEC61_*, Sec61-mCherry | GS115 *his4*::P*_GAL83_*, Gal83-GFP(*HIS4*) *SEC61*::P*_SEC61_*, Sec61-mCherry(*KAN*) | This study |
| Δ*nugM* + Gal83-GFP + Sec61-mCherry | SJCF2776 | SJCF2773 | Δ*nugM* + P*_GAL83_*, Gal83-GFP + P*_SEC61_*, Sec61-mCherry | GS115 Δ*nugM*::*NAT* *his4*::P*_GAL83_*, Gal83-GFP(*HIS4*) *SEC61*::P*_SEC61_*, Sec61-mCherry(*KAN*) | This study |
| WT + P*_MXR1_*, Mxr1^S215A^ | SJCF2750 | GS115 | WT + P*_MXR1_*, Mxr1^S215A^-HA | GS115 *his4*::P*_MXR1_*, Mxr1^S215A^-HA(*HIS4*) | This study |
| Δ*gal83* + P*_MXR1_*, Mxr1^S215A^ | SJCF2751 | Δ*PAS_chr1-4_0498* | Δ*gal83* + P*_MXR1_*, Mxr1^S215A^-HA | GS115 Δ*gal83*::*ZEO* *his4*::P*_MXR1_*, Mxr1^S215A^-HA(*HIS4*) | This study |
| Δ*nugM* + P*_MXR1_*, Mxr1^S215A^ | SJCF2752 | SJCF2750 | Δ*nugM* + P*_MXR1_*, Mxr1^S215A^-HA | GS115 Δ*nugM*::*NAT* *his4*::P*_MXR1_*, Mxr1^S215A^-HA(*HIS4*) | This study |
| Δ*nugM* + OE-Mit1 | SJCF2764 | WT-Mit1 | Δ*nugM* + P*_GAPDH_*, Mit1 | GS115 Δ*nugM*::*NAT* P*_GAPDH_*::pP6GM1(P*_GAPDH_*, Mit1; *HIS4*, *BLA*) | This study |
| Δ*gal83* + OE-Mit1 | SJCF2758 | WT-Mit1 | Δ*gal83* + P*_GAPDH_*, Mit1 | GS115 Δ*gal83*::*NAT* P*_GAPDH_*::pP6GM1(P*_GAPDH_*, Mit1; *HIS4*, *BLA*) | This study |
| WT | GS115 + pIB1 | GS115 | WT | GS115 *his4*::pIB1(empty plasmid; *HIS4*) | This study |
| Δ*gal83* | Δ*gal83* + pIB1 | Δ*PAS_chr1-4_0498* | Δ*gal83* | GS115 Δ*gal83*::*ZEO* *his4*::pIB1(empty plasmid; *HIS4*) | This study |
| Δ*nugM* | Δ*nugM* + pIB1 | SJCF2722 | Δ*nugM* | GS115 Δ*nugM*::*NAT* *his4*::pIB1(empty plasmid; *HIS4*) | This study |
| WT + SoNar | GS115 + SoNar | GS115 | GS115 + SoNar | GS115 *his4*::pIB2-SoNar(P*_GAPDH_*, SoNar; *HIS4*) | This study |
| Δ*gal83* + SoNar | Δ*gal83* + SoNar | Δ*PAS_chr1-4_0498* | Δ*gal83* + SoNar | GS115 Δ*gal83*::*ZEO* *his4*::pIB2-SoNar(P*_GAPDH_*, SoNar; *HIS4*) | This study |
| Δ*nugM* + SoNar | Δ*nugM* + SoNar | SJCF2722 | Δ*nugM* + SoNar | GS115 Δ*nugM*::*NAT* *his4*::pIB2-SoNar(P*_GAPDH_*, SoNar; *HIS4*) | This study |
| Δ*cat2* + SoNar | Δ*cat2* + SoNar | Δ*cat2* | Δ*cat2* + SoNar | GS115 Δ*cat2*::*NAT his4*::pIB2-SoNar(P*_GAPDH_*, SoNar; *HIS4*) | This study |
| Δ*pot1* + SoNar | Δ*pot1* + SoNar | Δ*pot1* | Δ*pot1* + SoNar | PPY12 Δ*pot1*::*ARG4* *his4*::pIB2-SoNar(P*_GAPDH_*, SoNar; *HIS4*) | This study |
| Δ*gpdA* Δ*mdhB* + SoNar | Δ*gpdA* Δ*mdhB* + SoNar | Δ*gpdA* Δ*mdhB* | Δ*gpdA* Δ*mdhB* + SoNar | GS115 Δ*gpdA*::*NAT* Δ*mdhB*::*HPH his4*::pIB2-SoNar(P*_GAPDH_*, SoNar; *HIS4*) | This study |
| WT + SoNar-ePTS1 | GS115 + SoNar-ePTS1 | GS115 | GS115 + SoNar-ePTS1 | GS115 *his4*::pIB2-SoNar-ePTS1(P*_GAPDH_*, SoNar-ePTS1; *HIS4*) | This study |
| Δ*gal83* + SoNar-ePTS1 | Δ*gal83* + SoNar-ePTS1 | Δ*PAS_chr1-4_0498* | Δ*gal83* + SoNar-ePTS1 | GS115 Δ*gal83*::*ZEO* *his4*::pIB2-SoNar-ePTS1(P*_GAPDH_*, SoNar-ePTS1; *HIS4*) | This study |
| Δ*nugM* + SoNar-ePTS1 | Δ*nugM* + SoNar-ePTS1 | SJCF2722 | Δ*nugM* + SoNar-ePTS1 | GS115 Δ*nugM*::*NAT* *his4*::pIB2-SoNar-ePTS1(P*_GAPDH_*, SoNar-ePTS1; *HIS4*) | This study |
| Δ*cat2* + SoNar-ePTS1 | Δ*cat2* + SoNar-ePTS1 | Δ*cat2* | Δ*cat2* + SoNar-ePTS1 | GS115 Δ*cat2*::*NAT his4*::pIB2-SoNar-ePTS1(P*_GAPDH_*, SoNar-ePTS1; *HIS4*) | This study |
| Δ*pot1* + SoNar-ePTS1 | Δ*pot1* + SoNar-ePTS1 | Δ*pot1* | Δ*pot1* + SoNar-ePTS1 | PPY12 Δ*pot1*::*ARG4* *his4*::pIB2-SoNar-ePTS1(P*_GAPDH_*, SoNar-ePTS1; *HIS4*) | This study |
| Δ*gpdA* Δ*mdhB* + SoNar-ePTS1 | Δ*gpdA* Δ*mdhB* + SoNar-ePTS1 | Δ*gpdA* Δ*mdhB* | Δ*gpdA* Δ*mdhB* + SoNar-ePTS1 | GS115 Δ*gpdA*::*NAT* Δ*mdhB*::*HPH his4*::pIB2-SoNar-ePTS1(P*_GAPDH_*, SoNar-ePTS1; *HIS4*) | This study |
| WT + cpYFP | GS115 + cpYFP | GS115 | GS115 + cpYFP | GS115 *his4*::pIB2-cpYFP(P*_GAPDH_*, cpYFP; *HIS4*) | This study |
| Δ*gal83* + cpYFP | Δ*gal83* + cpYFP | Δ*PAS_chr1-4_0498* | Δ*gal83* + cpYFP | GS115 Δ*gal83*::*ZEO* *his4*::pIB2-cpYFP(P*_GAPDH_*, cpYFP; *HIS4*) | This study |
| Δ*nugM* + cpYFP | Δ*nugM* + cpYFP | SJCF2722 | Δ*nugM* + cpYFP | GS115 Δ*nugM*::*NAT* *his4*::pIB2-cpYFP(P*_GAPDH_*, cpYFP; *HIS4*) | This study |
| Δ*cat2* + cpYFP | Δ*cat2* + cpYFP | Δ*cat2* | Δ*cat2* + cpYFP | GS115 Δ*cat2*::*NAT his4*::pIB2-cpYFP(P*_GAPDH_*, cpYFP; *HIS4*) | This study |
| Δ*pot1* + cpYFP | Δ*pot1* + cpYFP | Δ*pot1* | Δ*pot1* + cpYFP | PPY12 Δ*pot1*::*ARG4* *his4*::pIB2-cpYFP(P*_GAPDH_*, cpYFP; *HIS4*) | This study |
| Δ*gpdA* Δ*mdhB* + cpYFP | Δ*gpdA* Δ*mdhB* + cpYFP | Δ*gpdA* Δ*mdhB* | Δ*gpdA* Δ*mdhB* + cpYFP | GS115 Δ*gpdA*::*NAT* Δ*mdhB*::*HPH his4*::pIB2-cpYFP(P*_GAPDH_*, cpYFP; *HIS4*) | This study |
| WT + cpYFP-ePTS1 | GS115 + cpYFP-ePTS1 | GS115 | GS115 + cpYFP-ePTS1 | GS115 *his4*::pIB2-cpYFP-ePTS1(P*_GAPDH_*, cpYFP-ePTS1; *HIS4*) | This study |
| Δ*gal83* + cpYFP-ePTS1 | Δ*gal83* + cpYFP-ePTS1 | Δ*PAS_chr1-4_0498* | Δ*gal83* + cpYFP-ePTS1 | GS115 Δ*gal83*::*ZEO* *his4*::pIB2-cpYFP-ePTS1(P*_GAPDH_*, cpYFP-ePTS1; *HIS4*) | This study |
| Δ*nugM* + cpYFP-ePTS1 | Δ*nugM* + cpYFP-ePTS1 | SJCF2722 | Δ*nugM* + cpYFP-ePTS1 | GS115 Δ*nugM*::*NAT* *his4*::pIB2-cpYFP-ePTS1(P*_GAPDH_*, cpYFP-ePTS1; *HIS4*) | This study |
| Δ*cat2* + cpYFP-ePTS1 | Δ*cat2* + cpYFP-ePTS1 | Δ*cat2* | Δ*cat2* + cpYFP-ePTS1 | GS115 Δ*cat2*::*NAT his4*::pIB2-cpYFP-ePTS1(P*_GAPDH_*, cpYFP-ePTS1; *HIS4*) | This study |
| Δ*pot1* + cpYFP-ePTS1 | Δ*pot1* + cpYFP-ePTS1 | Δ*pot1* | Δ*pot1* + cpYFP-ePTS1 | PPY12 Δ*pot1*::*ARG4* *his4*::pIB2-cpYFP-ePTS1(P*_GAPDH_*, cpYFP-ePTS1; *HIS4*) | This study |
| Δ*gpdA* Δ*mdhB* + cpYFP-ePTS1 | Δ*gpdA* Δ*mdhB* + cpYFP-ePTS1 | Δ*gpdA* Δ*mdhB* | Δ*gpdA* Δ*mdhB* + cpYFP-ePTS1 | GS115 Δ*gpdA*::*NAT* Δ*mdhB*::*HPH his4*::pIB2-cpYFP-ePTS1(P*_GAPDH_*, cpYFP-ePTS1; *HIS4*) | This study |

Table S1. Strains and plasmids.

**References**

1. Cregg JM, Barringer KJ, Hessler AY, Madden KR (1985) *Pichia pastoris* as a host system for transformations. *Mol Cell Biol* **5**: 3376-85

2. Gould SJ, McCollum D, Spong AP, Heyman JA, Subramani S (1992) Development of the yeast *Pichia pastoris* as a model organism for a genetic and molecular analysis of peroxisome assembly. *Yeast* **8**: 613-28

3. Cregg J, Madden KR (1987) *Development of yeast transformation systems and construction of methanol-utilization-defective mutants of Pichia pastoris by gene disruption.* In Biological research on industrial yeasts, Stewart GG, Russell RD, Klein RD, Hiebesch RR (eds) pp 1-18. Boca Raton, Florida: CRC Press, inc.

4. Shen W, Kong C, Xue Y, Liu Y, Cai M, Zhang Y, Jiang T, Zhou X, Zhou M (2016) Kinase Screening in *Pichia pastoris* Identified Promising Targets Involved in Cell Growth and Alcohol Oxidase 1 Promoter (PAOX1) Regulation. *PLoS One* **11**: e0167766

5. Wang J, Wang X, Shi L, Qi F, Zhang P, Zhang Y, Zhou X, Song Z, Cai M (2017) Methanol-Independent Protein Expression by AOX1 Promoter with trans-Acting Elements Engineering and Glucose-Glycerol-Shift Induction in *Pichia pastoris*. *Sci Rep* **7**: 41850

6. Johnson MA, Waterham HR, Ksheminska GP, Fayura LR, Cereghino JL, Stasyk OV, Veenhuis M, Kulachkovsky AR, Sibirny AA, Cregg JM (1999) Positive selection of novel peroxisome biogenesis-defective mutants of the yeast *Pichia pastoris*. *Genetics* **151**: 1379-91

7. Johnson MA, Snyder WB, Cereghino JL, Veenhuis M, Subramani S, Cregg JM (2001) *Pichia pastoris* Pex14p, a phosphorylated peroxisomal membrane protein, is part of a PTS-receptor docking complex and interacts with many peroxins. *Yeast* **18**: 621-41

8. Cregg JM, Madden KR, Barringer KJ, Thill GP, Stillman CA (1989) Functional characterization of the two alcohol oxidase genes from the yeast *Pichia pastoris*. *Mol Cell Biol* **9**: 1316-23

9. McCollum D, Monosov E, Subramani S (1993) The pas8 mutant of *Pichia pastoris* exhibits the peroxisomal protein import deficiencies of Zellweger syndrome cells--the PAS8 protein binds to the COOH-terminal tripeptide peroxisomal targeting signal, and is a member of the TPR protein family. *J Cell Biol* **121**: 761-74

10. Elgersma Y, Elgersma-Hooisma M, Wenzel T, McCaffery JM, Farquhar MG, Subramani S (1998) A mobile PTS2 receptor for peroxisomal protein import in *Pichia pastoris*. *J Cell Biol* **140**: 807-20
