# Supplementary material for: OXPHOS deficiencies affect peroxisome proliferation by downregulating genes controlled by the SNF1 signaling pathway
Source: eLife. 2022 Apr 25;11:e75143. doi: 10.7554/eLife.75143 (PMC9094750; doi:10.7554/eLife.75143)
Supplement: Supplementary file 2. [file elife-75143-supp2.docx]

| **Name** | **Primer** | **Target** |
| --- | --- | --- |
| 18s-rRNA-qF | GAGGATTGACAGGATGAGAGC | 18S ribosomal RNA |
| 18s-rRNA-qR | CAAGGTCTCGTTCGTTATCGC | 18S ribosomal RNA |
| Pex11-qPCR-S | GAACAGGAAGGCTCTGAGATT | *PEX11* |
| PEX11-qPCR-AS | GGTGACCTTGTCGGTTAGTT | *PEX11* |
| AOX-qPCR-s | TCCAGAGGTTCCATTCACATTAC | *AOX1* |
| AOX-qPCR-as | CTTGTAAGCCCAAACCATAGGA | *AOX1* |
| POT1-qPCR-as | CTTCATCCTGGTCCACAGTAATAG | *POT1* |
| POT1-qPCR-s | GAGGAGATTATTCCCATCCAAGTAG | *POT1* |

Table S2. RT-qPCR primers.
