# Supplementary figures and images for: OXPHOS deficiencies affect peroxisome proliferation by downregulating genes controlled by the SNF1 signaling pathway (part 2 of 2)
Source: eLife. 2022 Apr 25;11:e75143. doi: 10.7554/eLife.75143 (PMC9094750; doi:10.7554/eLife.75143)

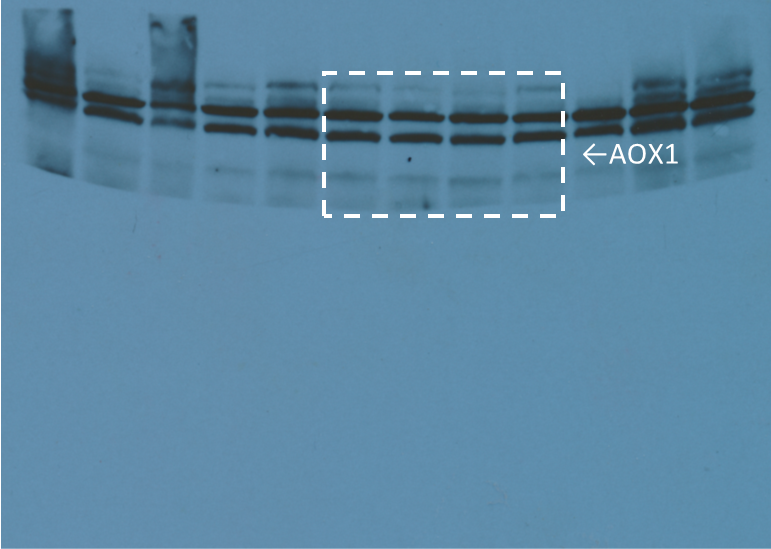

Supplement: Source data 1. [file elife-75143-data1.zip › Fig. 4C +glucose - Aox1_Annotated.tif]

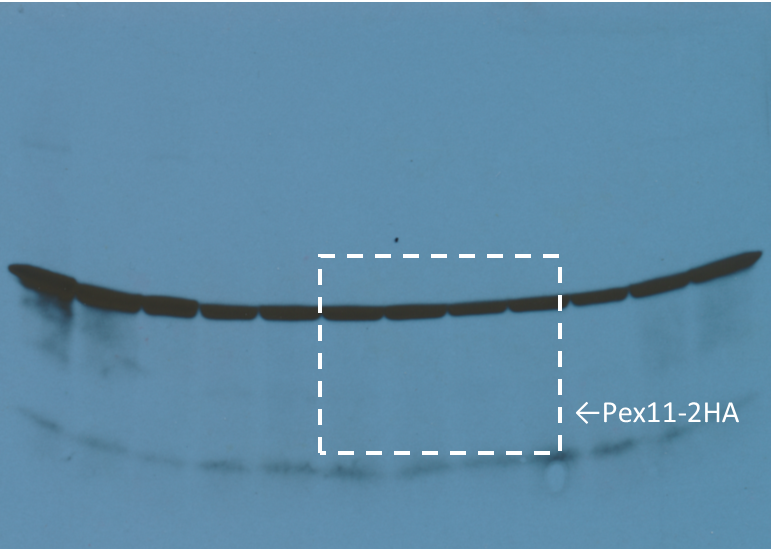

Supplement: Source data 1. [file elife-75143-data1.zip › Fig. 4C +glucose - HA_Annotated.tif]

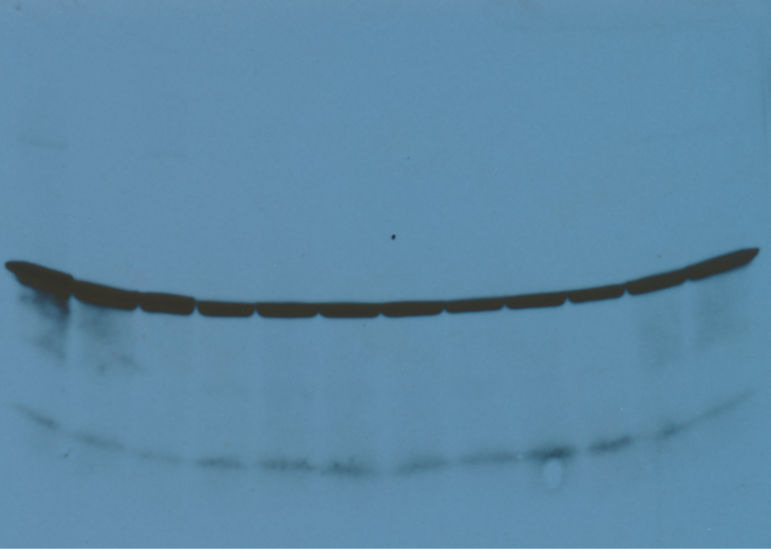

Supplement: Source data 1. [file elife-75143-data1.zip › Fig. 4C +glucose - HA_Raw.tif]

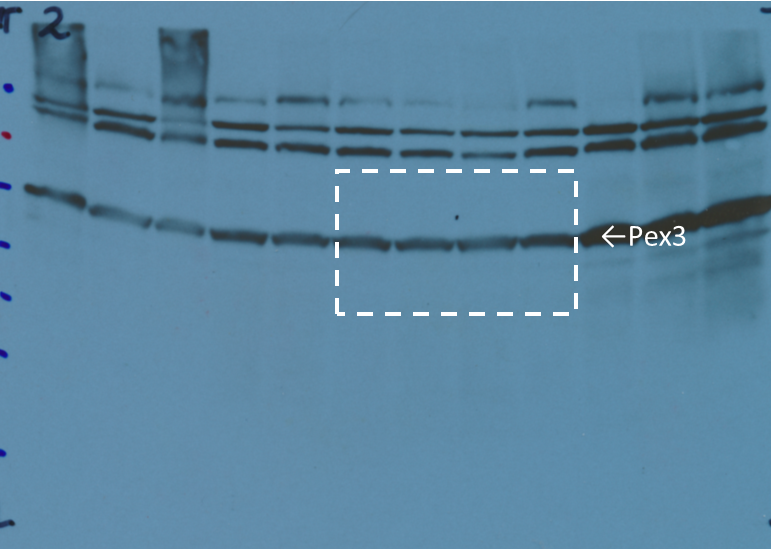

Supplement: Source data 1. [file elife-75143-data1.zip › Fig. 4C +glucose - Pex3_Annotated.tif]

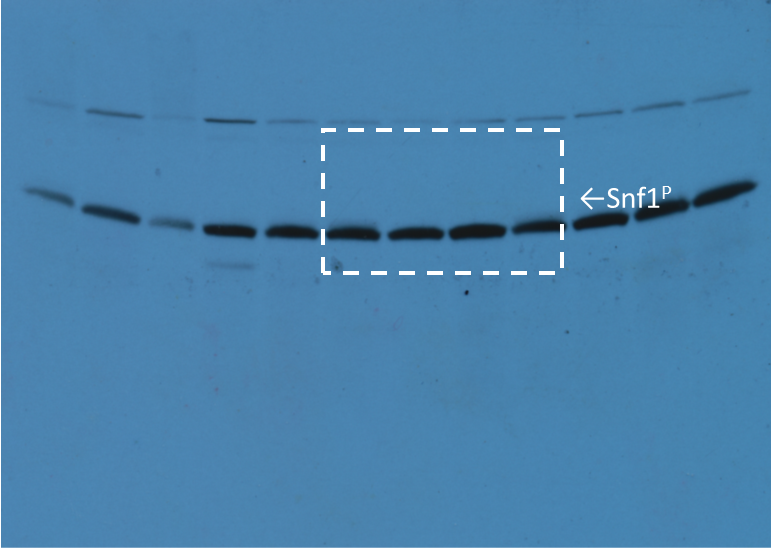

Supplement: Source data 1. [file elife-75143-data1.zip › Fig. 4C +glucose - Phospho Snf1_Annotated.tif]

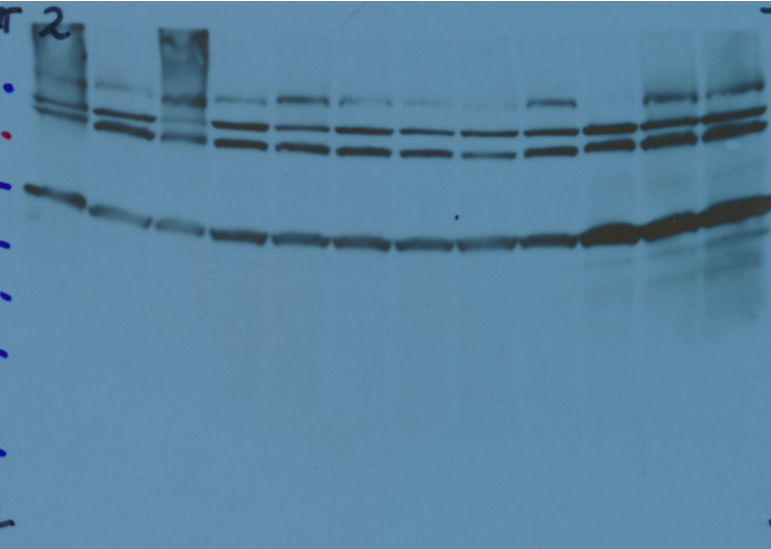

Supplement: Source data 1. [file elife-75143-data1.zip › Fig. 4C +glucose - Pex3_Raw.tif]

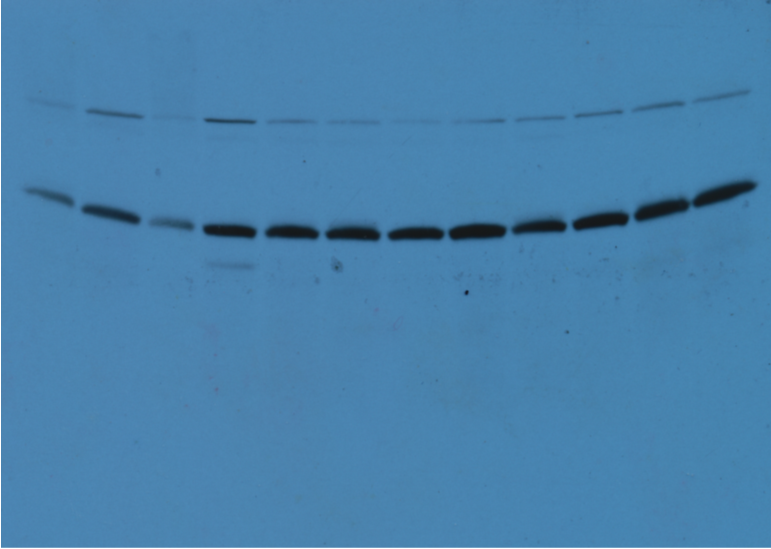

Supplement: Source data 1. [file elife-75143-data1.zip › Fig. 4C +glucose - Phospho Snf1_Raw.tif]

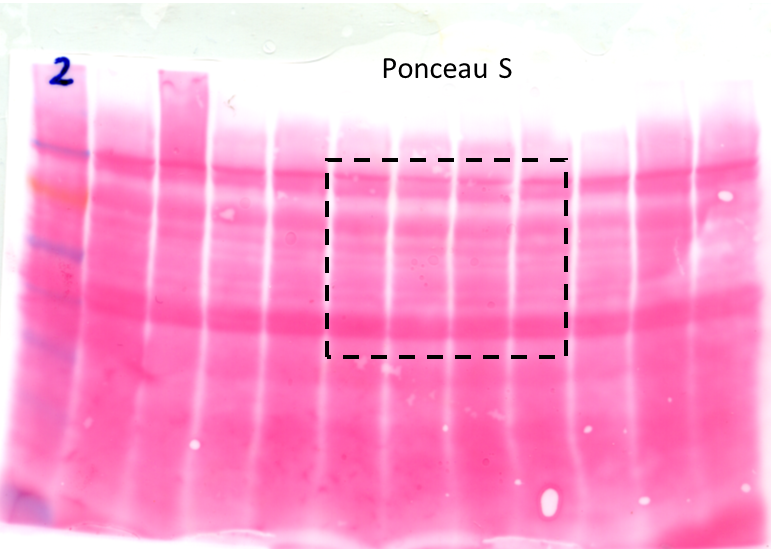

Supplement: Source data 1. [file elife-75143-data1.zip › Fig. 4C +glucose - Ponceau S_Annotated.tif]

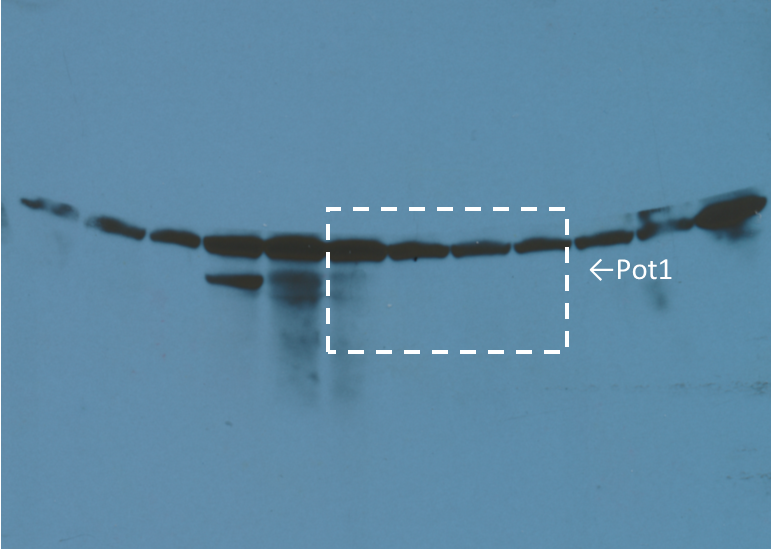

Supplement: Source data 1. [file elife-75143-data1.zip › Fig. 4C +glucose - Pot1_Annotated.tif]

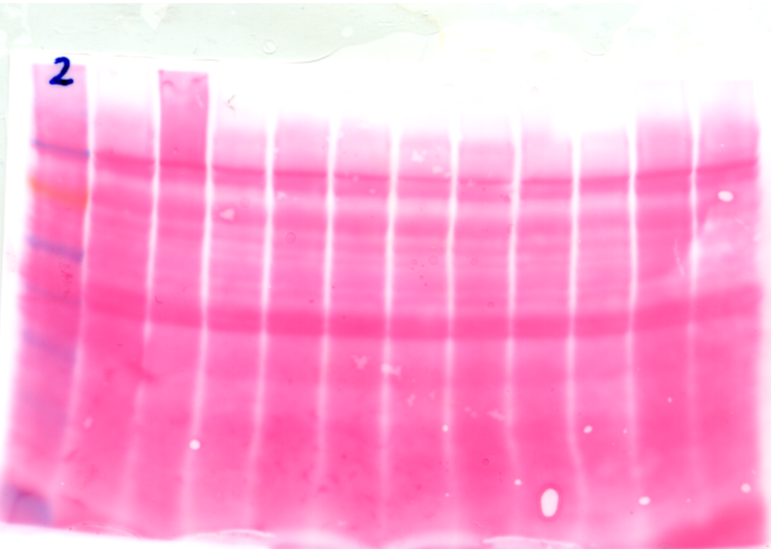

Supplement: Source data 1. [file elife-75143-data1.zip › Fig. 4C +glucose - Ponceau S_Raw.tif]

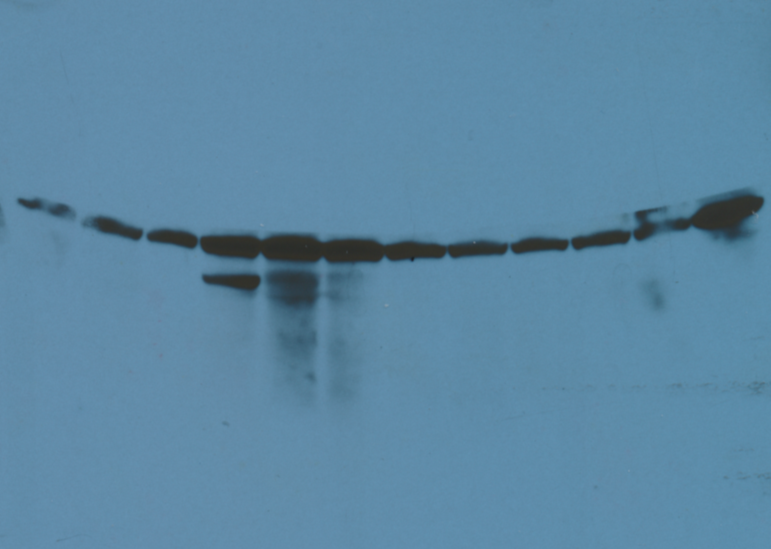

Supplement: Source data 1. [file elife-75143-data1.zip › Fig. 4C +glucose - Pot1_Raw.tif]

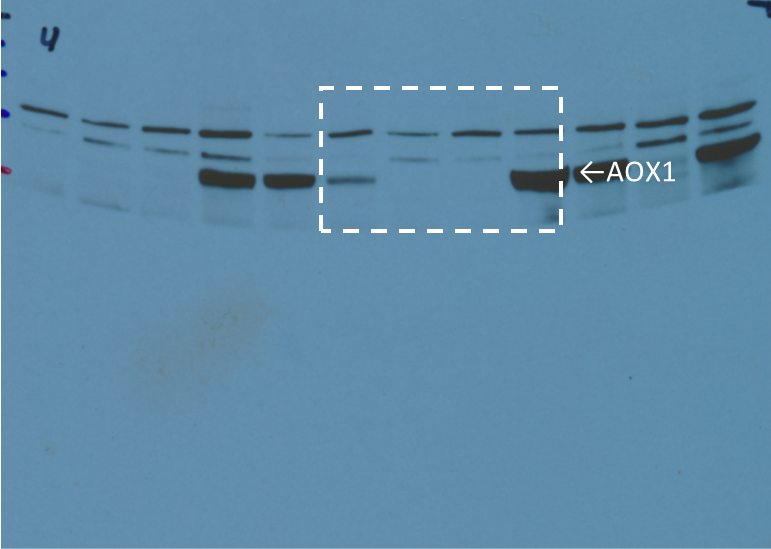

Supplement: Source data 1. [file elife-75143-data1.zip › Fig. 4C +methanol - Aox1_Annotated.tif]

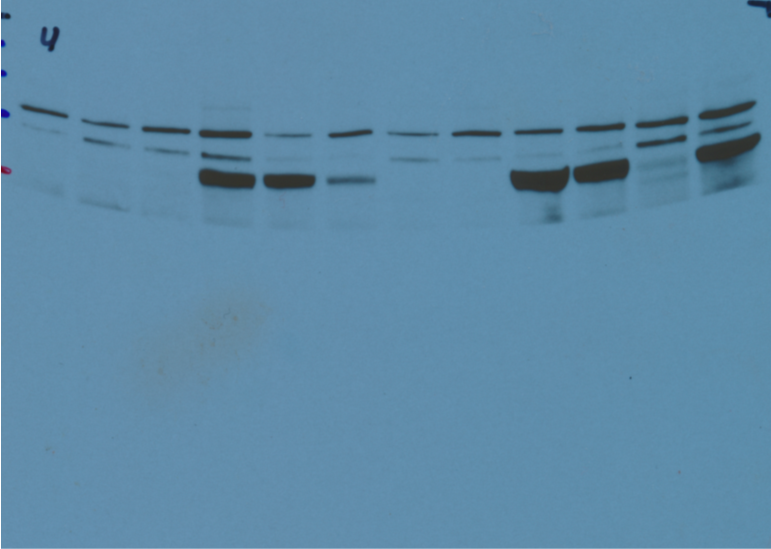

Supplement: Source data 1. [file elife-75143-data1.zip › Fig. 4C +methanol - Aox1_Raw.tif]

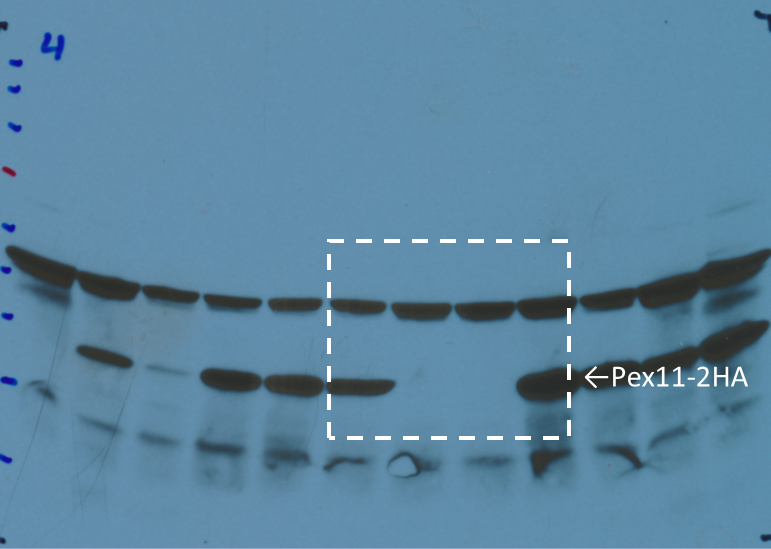

Supplement: Source data 1. [file elife-75143-data1.zip › Fig. 4C +methanol - HA_Annotated.tif]

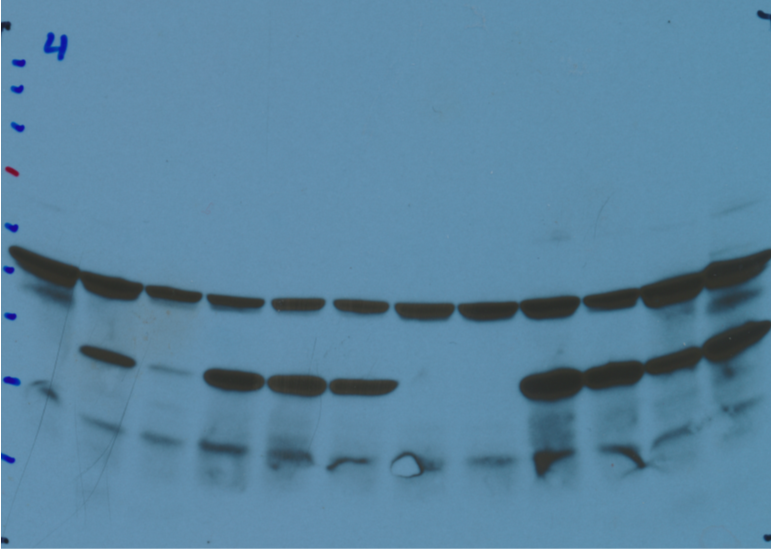

Supplement: Source data 1. [file elife-75143-data1.zip › Fig. 4C +methanol - HA_Raw.tif]

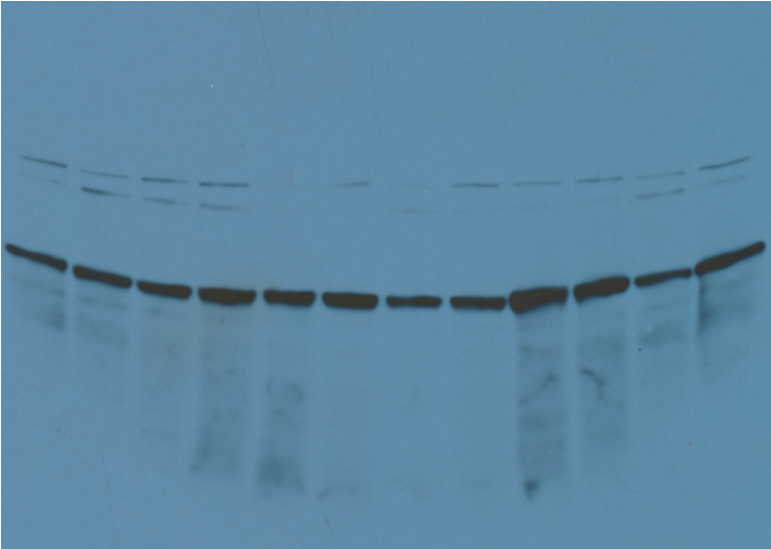

Supplement: Source data 1. [file elife-75143-data1.zip › Fig. 4C +methanol - Pex3_Raw.tif]

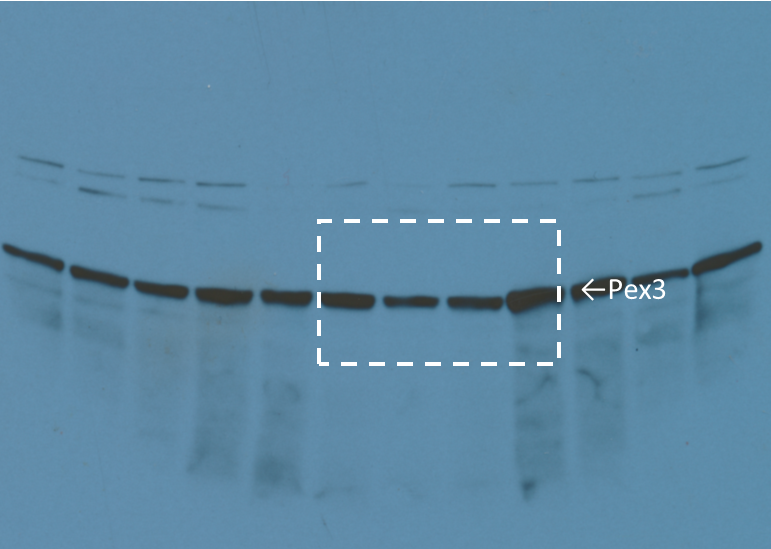

Supplement: Source data 1. [file elife-75143-data1.zip › Fig. 4C +methanol - Pex3_Annotated.tif]

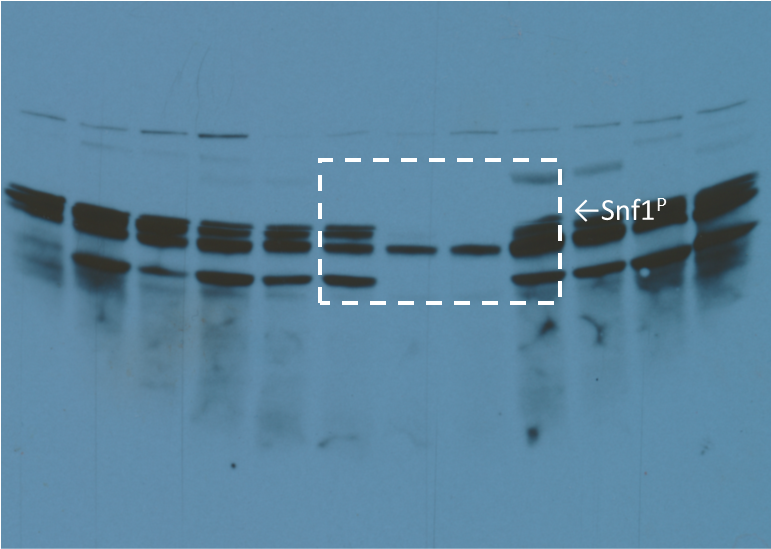

Supplement: Source data 1. [file elife-75143-data1.zip › Fig. 4C +methanol - Phospho Snf1_Annotated.tif]

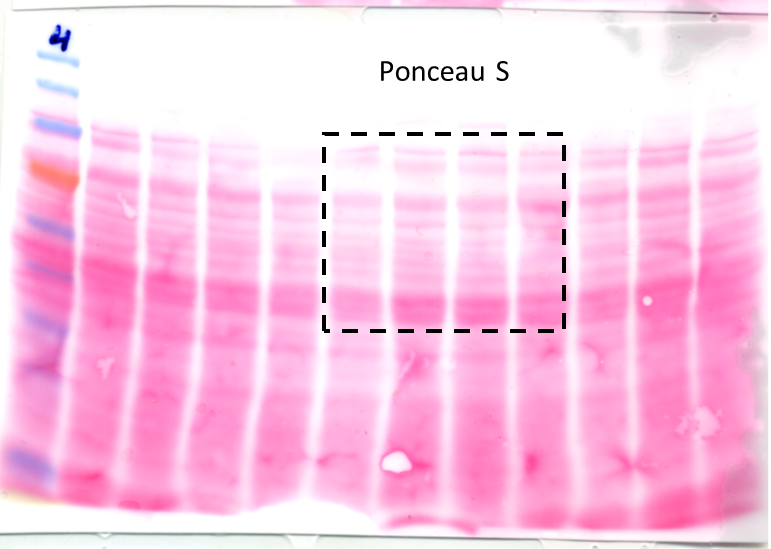

Supplement: Source data 1. [file elife-75143-data1.zip › Fig. 4C +methanol - Ponceau S_Annotated.tif]

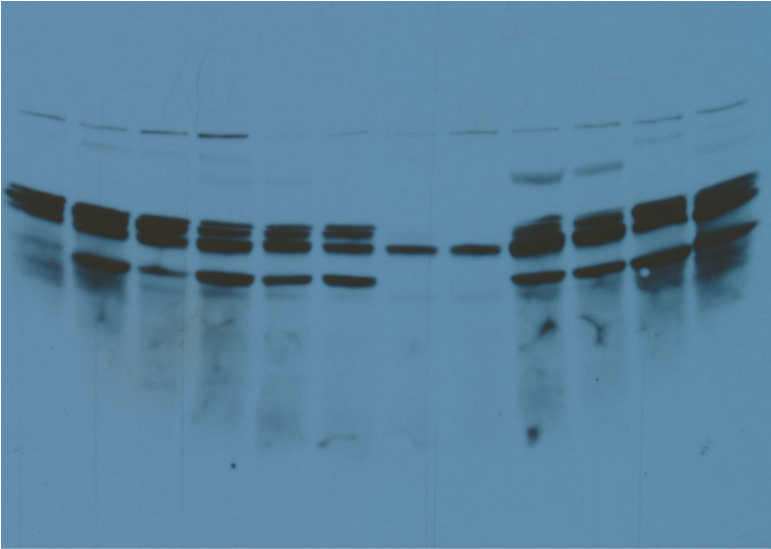

Supplement: Source data 1. [file elife-75143-data1.zip › Fig. 4C +methanol - Phospho Snf1_Raw.tif]

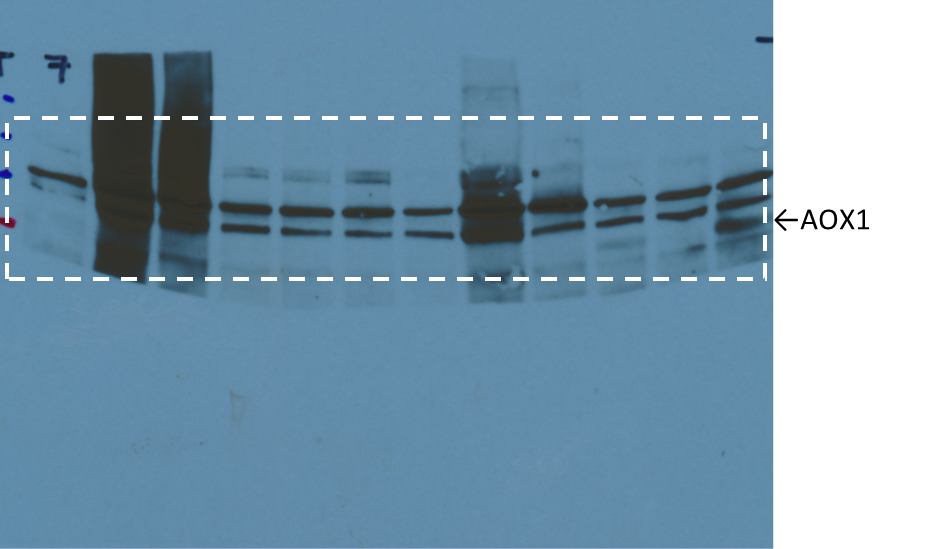

Supplement: Source data 2. [file elife-75143-data2.zip › Fig. 4D - Aox1_Annotated.tif]

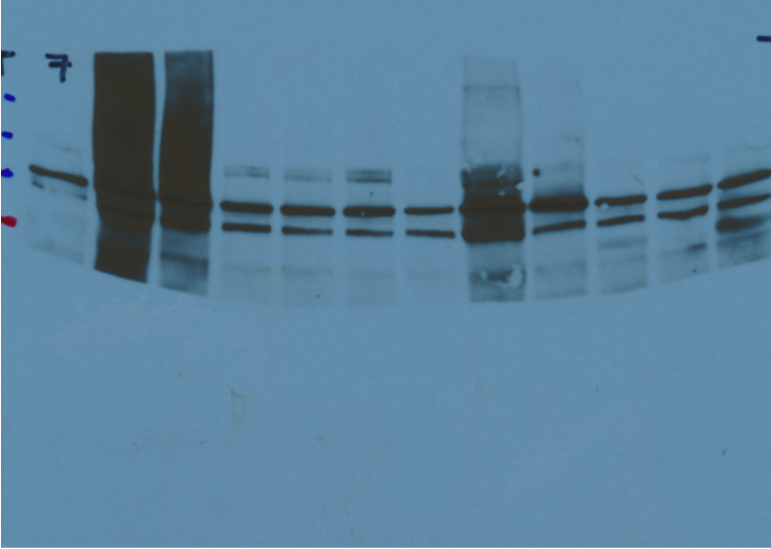

Supplement: Source data 2. [file elife-75143-data2.zip › Fig. 4D - Aox1_Raw.tif]

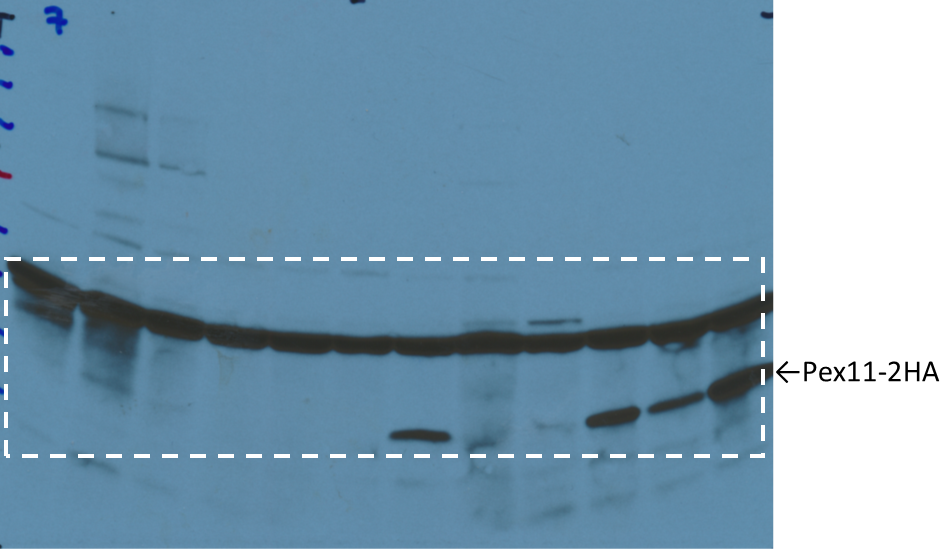

Supplement: Source data 2. [file elife-75143-data2.zip › Fig. 4D - HA_Annotated.tif]

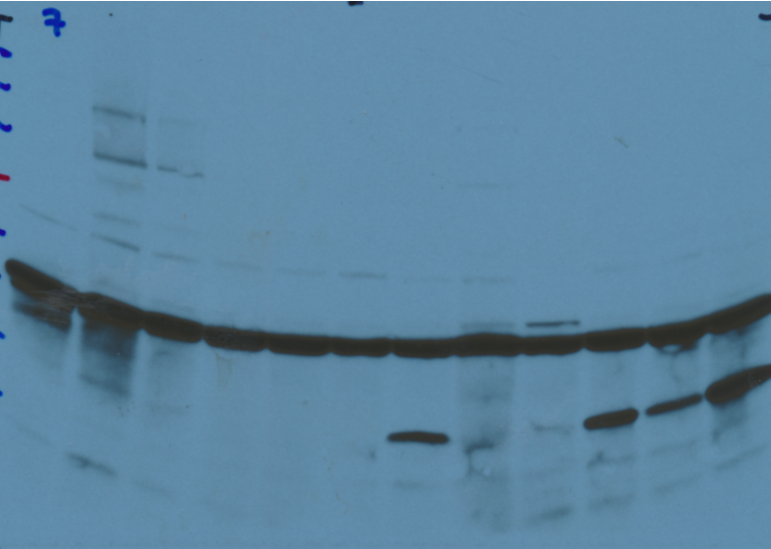

Supplement: Source data 2. [file elife-75143-data2.zip › Fig. 4D - HA_Raw.tif]

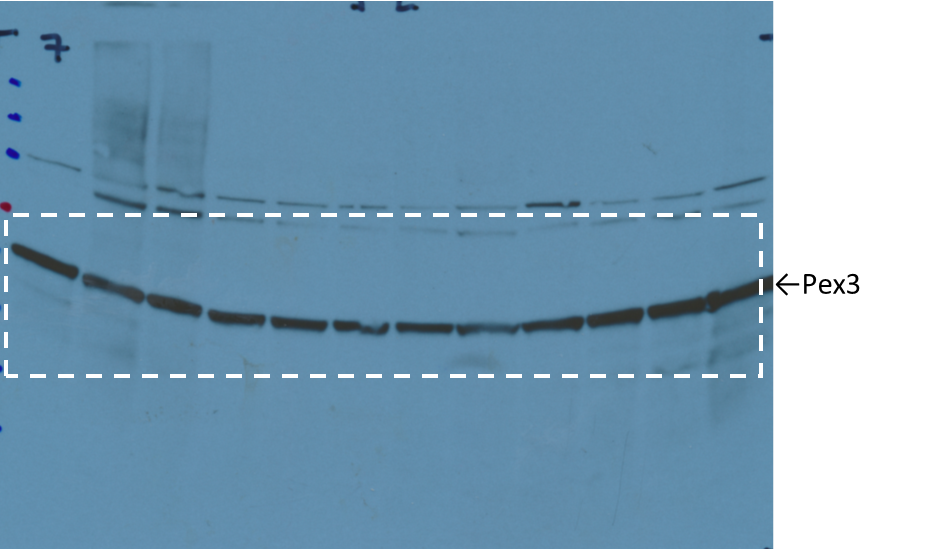

Supplement: Source data 2. [file elife-75143-data2.zip › Fig. 4D - Pex3_Annotated.tif]

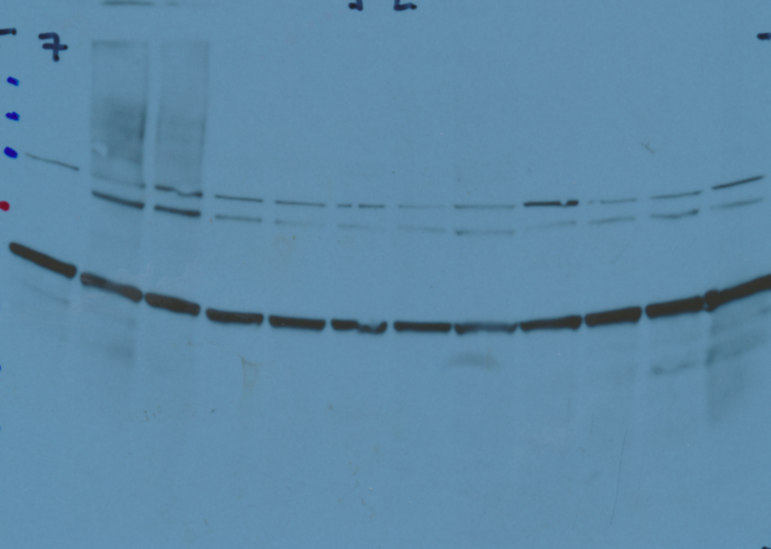

Supplement: Source data 2. [file elife-75143-data2.zip › Fig. 4D - Pex3_Raw.tif]

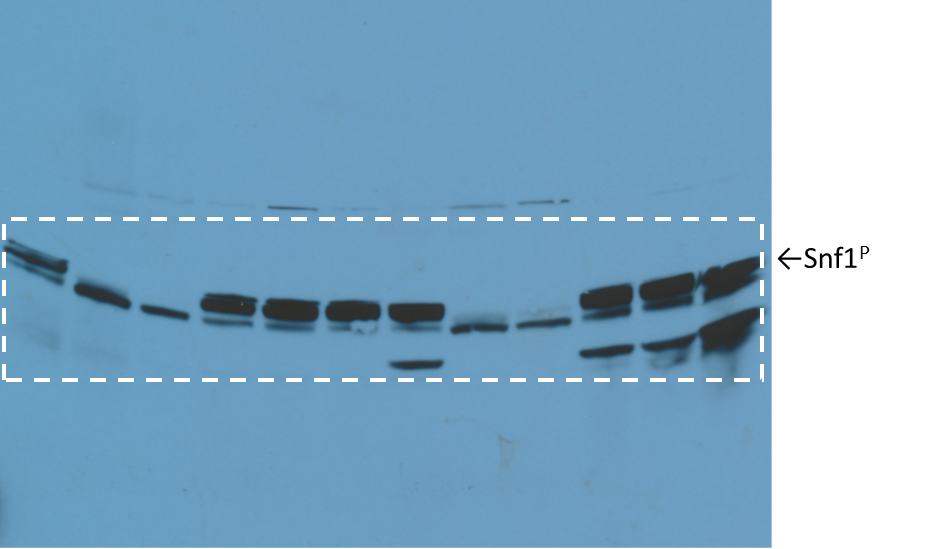

Supplement: Source data 2. [file elife-75143-data2.zip › Fig. 4D - Phospho Snf1_Annotated.tif]

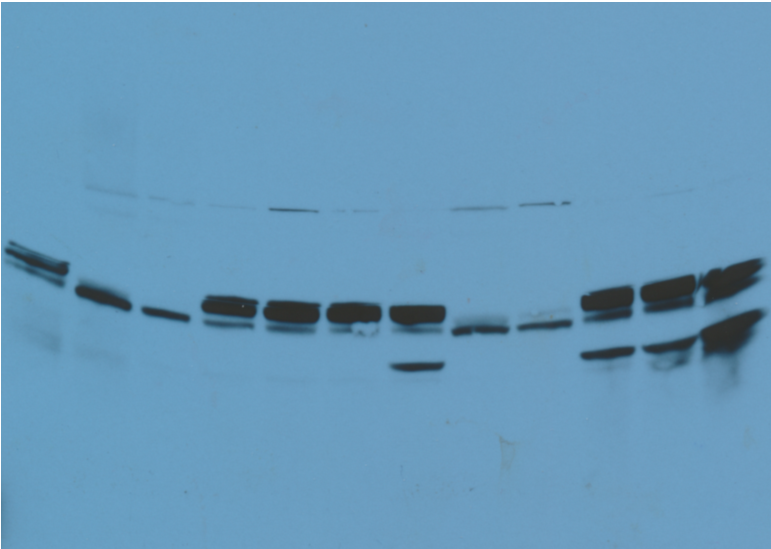

Supplement: Source data 2. [file elife-75143-data2.zip › Fig. 4D - Phospho Snf1_Raw.tif]

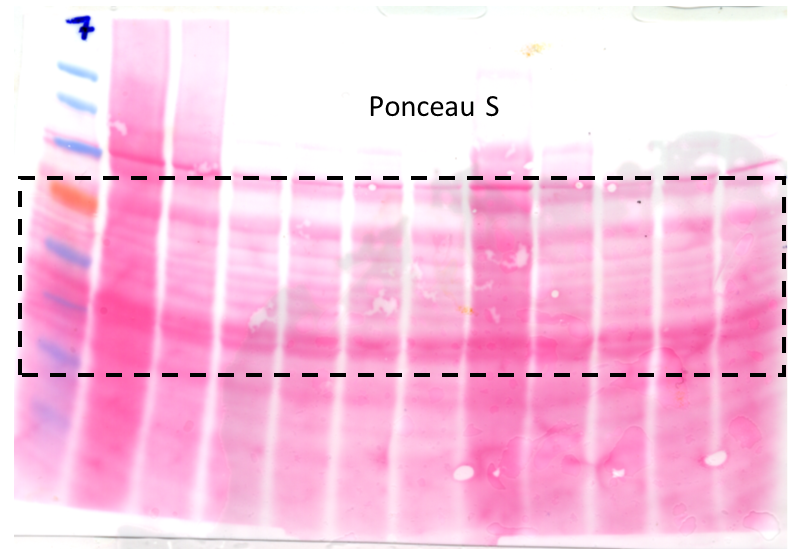

Supplement: Source data 2. [file elife-75143-data2.zip › Fig. 4D - Ponceau S_Annotated.tif]

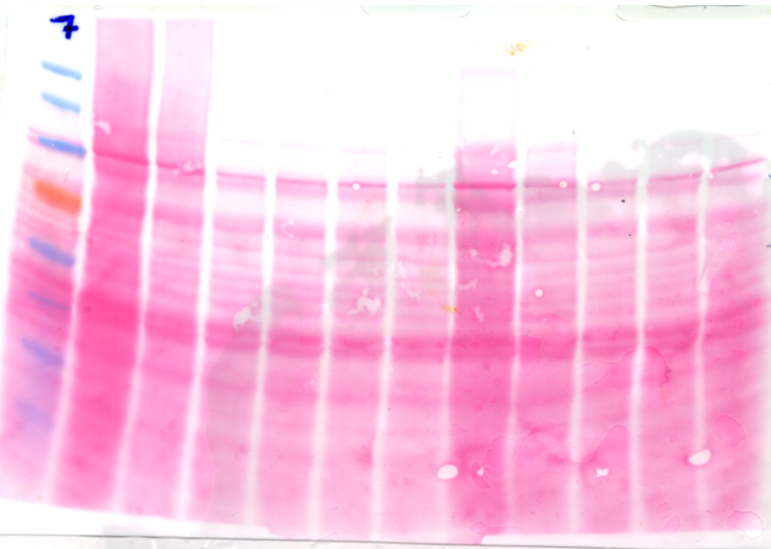

Supplement: Source data 2. [file elife-75143-data2.zip › Fig. 4D - Ponceau S_Raw.tif]

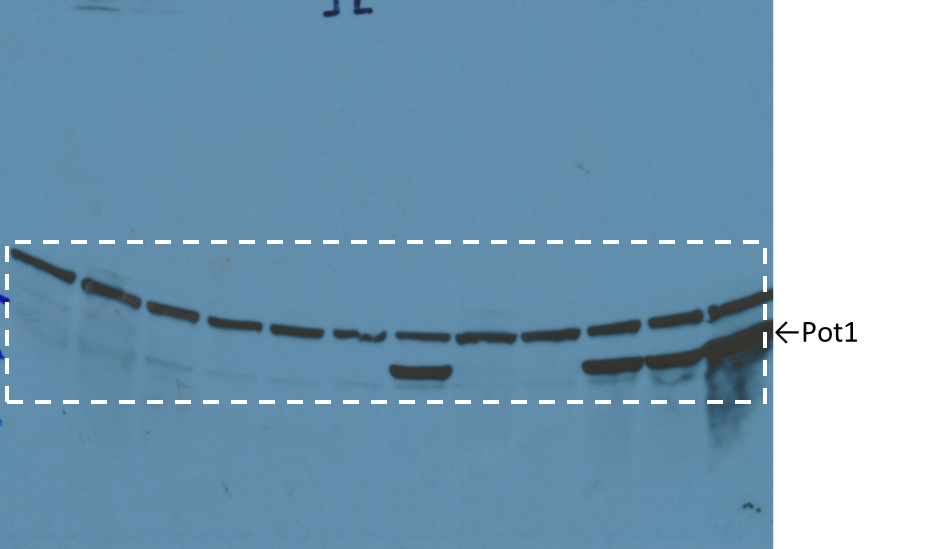

Supplement: Source data 2. [file elife-75143-data2.zip › Fig. 4D - Pot1_Annotated.tif]

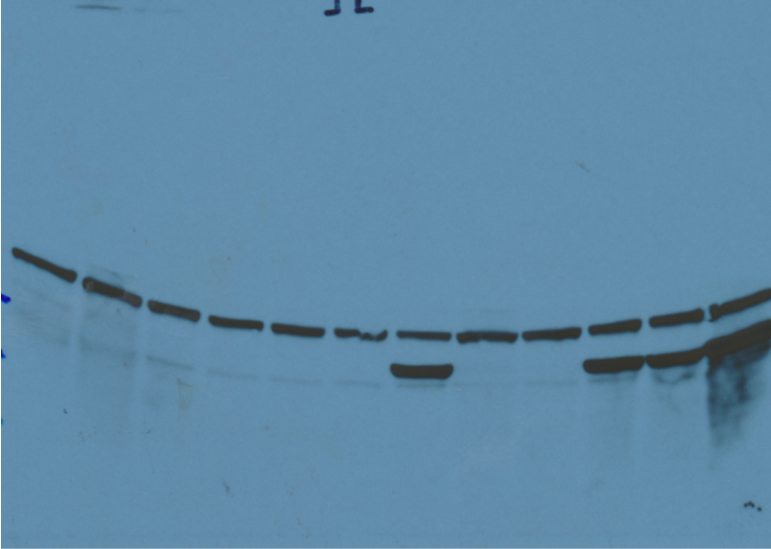

Supplement: Source data 2. [file elife-75143-data2.zip › Fig. 4D - Pot1_Raw.tif]

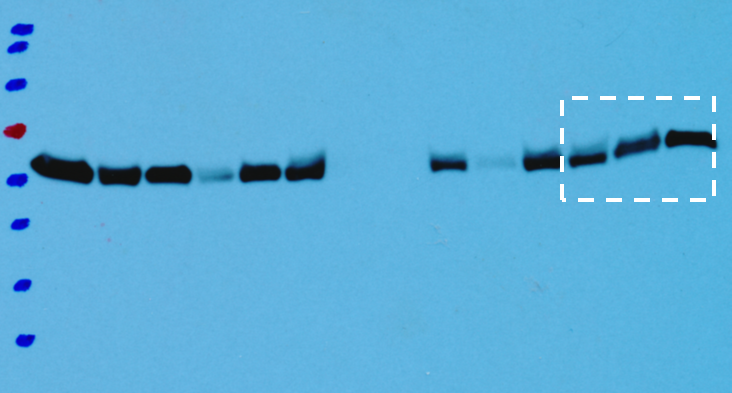

Supplement: Source data 2. [file elife-75143-data2.zip › Fig. 6A +methanol - Phospho Snf1_Annotated.tif]

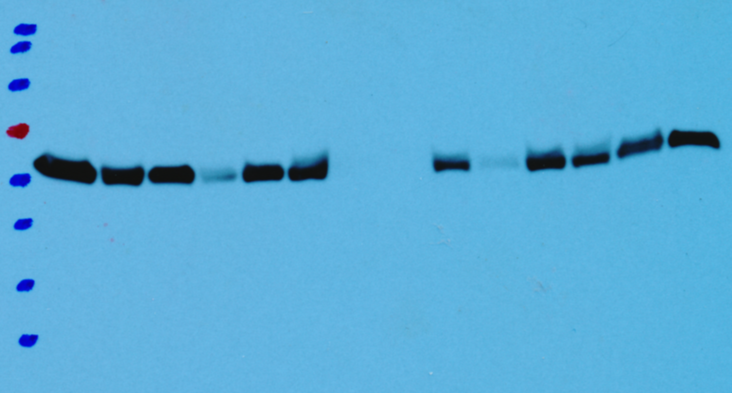

Supplement: Source data 2. [file elife-75143-data2.zip › Fig. 6A +methanol - Phospho Snf1_Raw.tif]

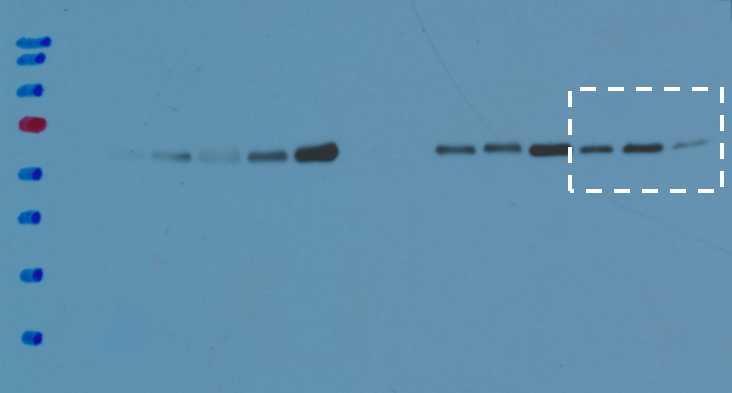

Supplement: Source data 2. [file elife-75143-data2.zip › Fig. 6A +oleate - Phospho Snf1_Annotated.tif]

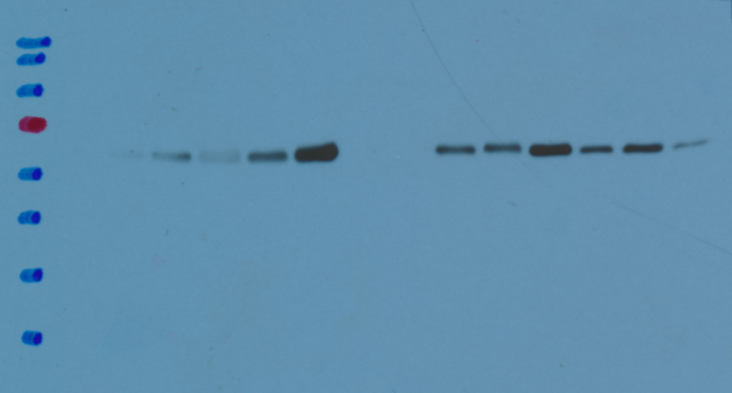

Supplement: Source data 2. [file elife-75143-data2.zip › Fig. 6A +oleate - Phospho Snf1_Raw.tif]

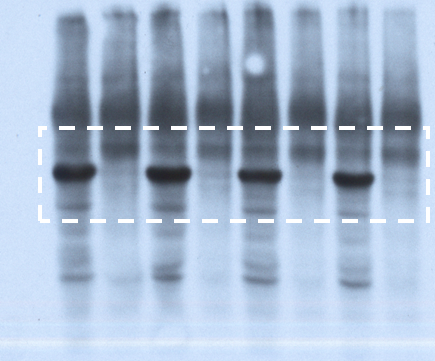

Supplement: Source data 2. [file elife-75143-data2.zip › Fig. 6B_Figure supplement 1 +methanol -Aox1_Annotated.tif]

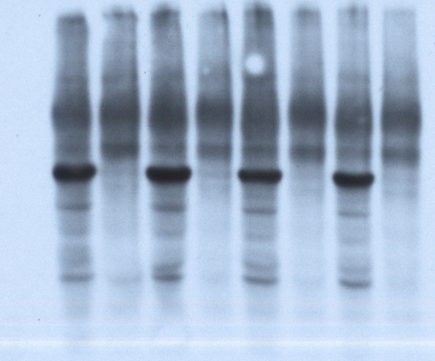

Supplement: Source data 2. [file elife-75143-data2.zip › Fig. 6B_Figure supplement 1 +methanol -Aox1_Raw.tif]

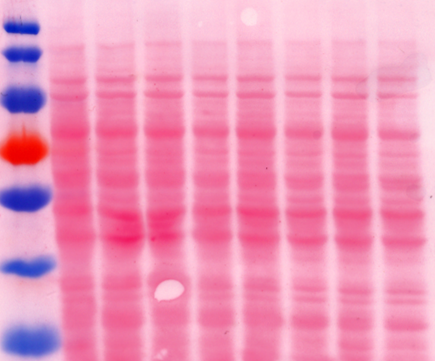

Supplement: Source data 2. [file elife-75143-data2.zip › Fig. 6B_Figure supplement 1 +methanol -Ponceau S_Raw.tif]

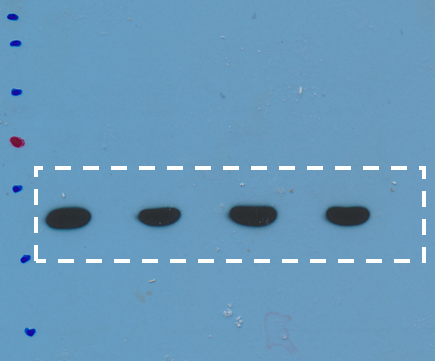

Supplement: Source data 2. [file elife-75143-data2.zip › Fig. 6B_Figure supplement 1 +methanol -Pot1_Annotated.tif]

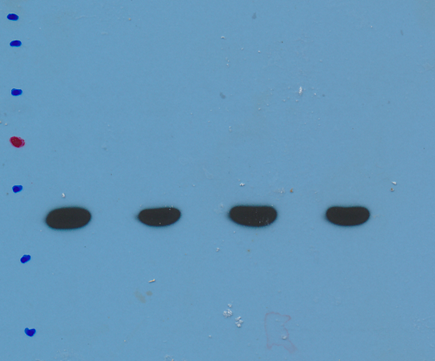

Supplement: Source data 2. [file elife-75143-data2.zip › Fig. 6B_Figure supplement 1 +methanol -Pot1_Raw.tif]

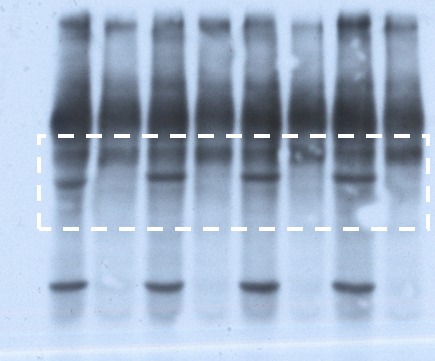

Supplement: Source data 2. [file elife-75143-data2.zip › Fig. 6B_Figure supplement 1 +oleate -Aox1_Annotated.tif]

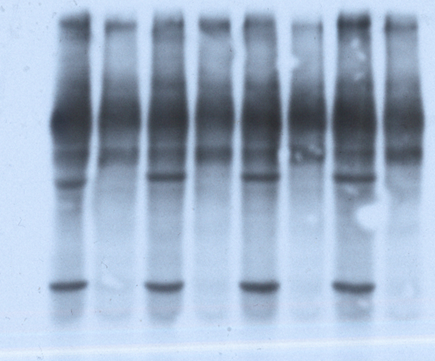

Supplement: Source data 2. [file elife-75143-data2.zip › Fig. 6B_Figure supplement 1 +oleate -Aox1_Raw.tif]

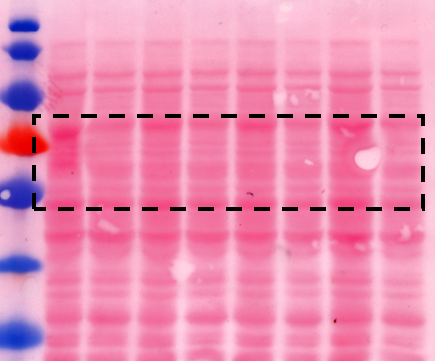

Supplement: Source data 2. [file elife-75143-data2.zip › Fig. 6B_Figure supplement 1 +oleate -Ponceau S_Annotated.tif]

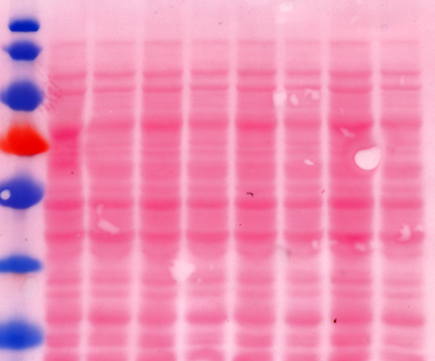

Supplement: Source data 2. [file elife-75143-data2.zip › Fig. 6B_Figure supplement 1 +oleate -Ponceau S_Raw.tif]

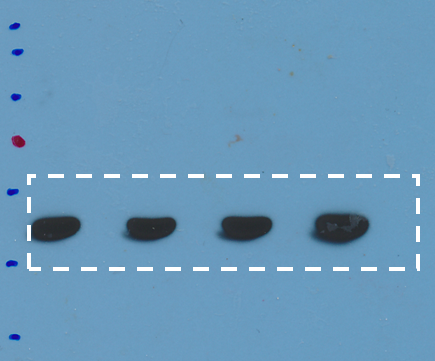

Supplement: Source data 2. [file elife-75143-data2.zip › Fig. 6B_Figure supplement 1 +oleate -Pot1_Annotated.tif]

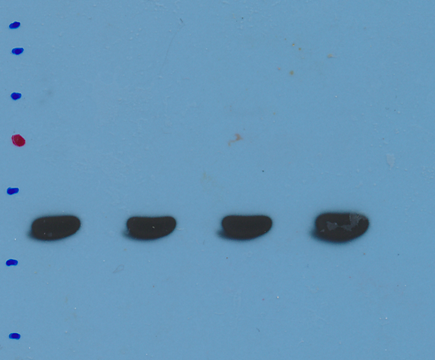

Supplement: Source data 2. [file elife-75143-data2.zip › Fig. 6B_Figure supplement 1 +oleate -Pot1_Raw.tif]

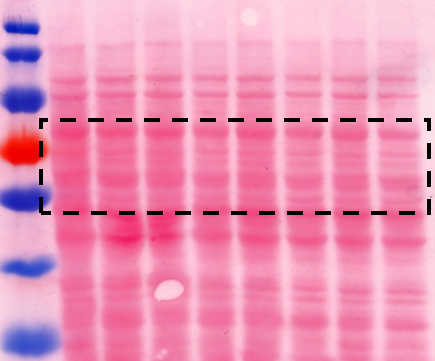

Supplement: Source data 2. [file elife-75143-data2.zip › Fig. 6B_Figure supplement 1+methanol -Ponceau S_Annotated.tif]

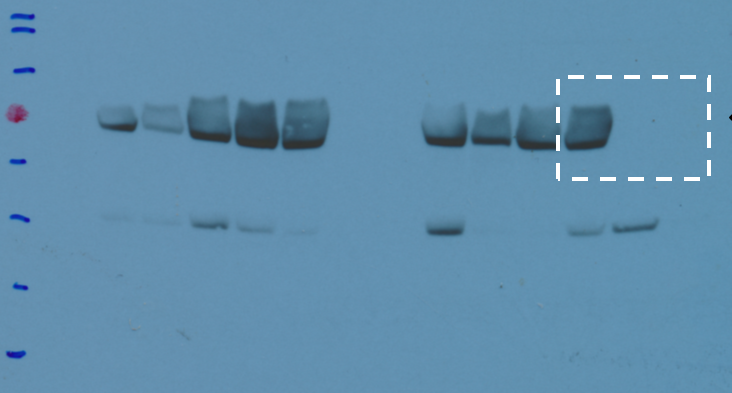

Supplement: Source data 2. [file elife-75143-data2.zip › Fig.6A +methanol - Aox1_Annotated.tif]

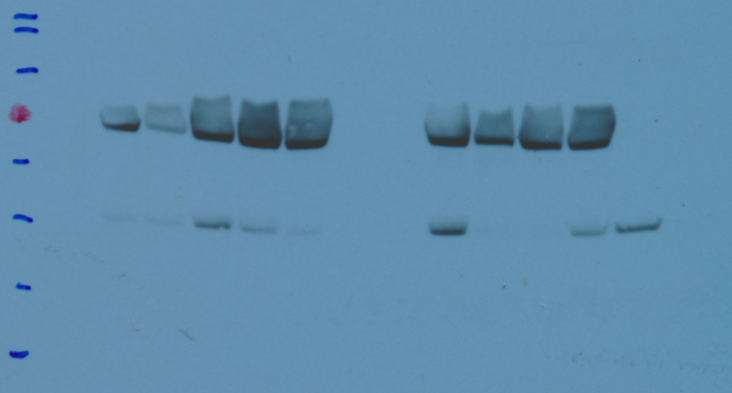

Supplement: Source data 2. [file elife-75143-data2.zip › Fig.6A +methanol - Aox1_Raw.tif]

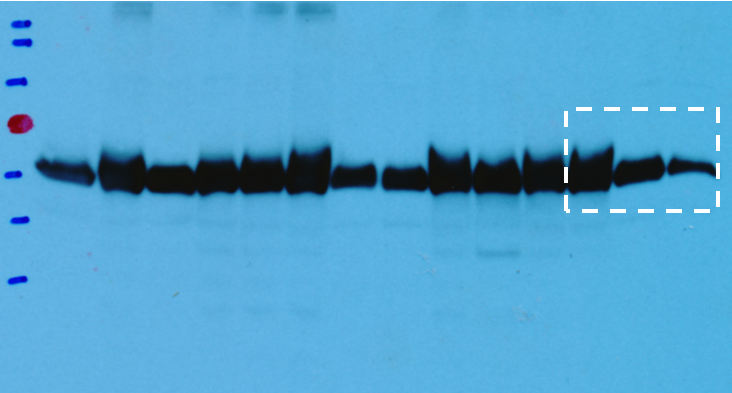

Supplement: Source data 2. [file elife-75143-data2.zip › Fig.6A +methanol - Pex3_Annotated.tif]

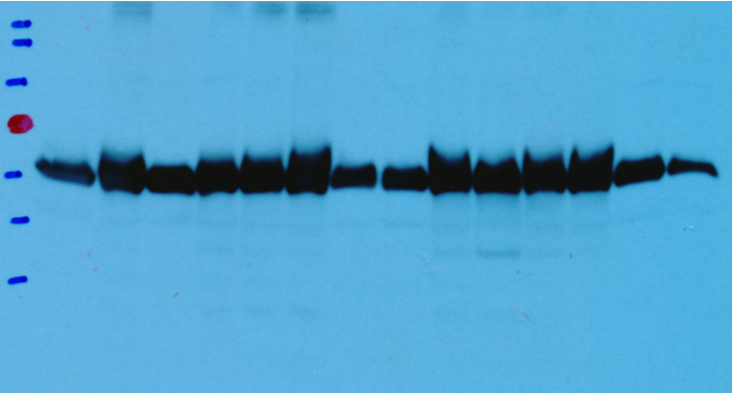

Supplement: Source data 2. [file elife-75143-data2.zip › Fig.6A +methanol - Pex3_Raw.tif]

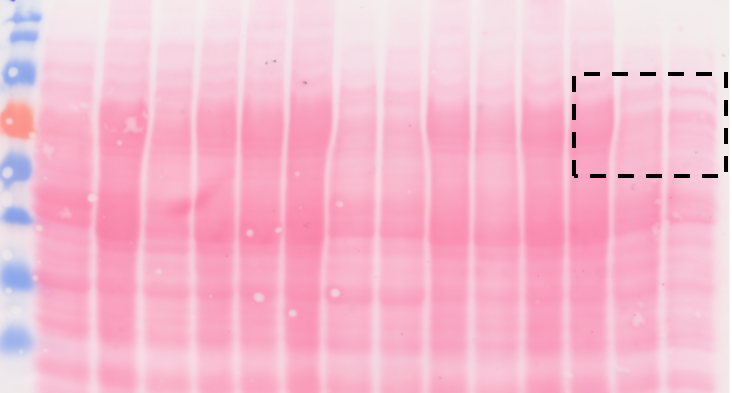

Supplement: Source data 2. [file elife-75143-data2.zip › Fig.6A +methanol - Ponceau S_Annotated.tif]

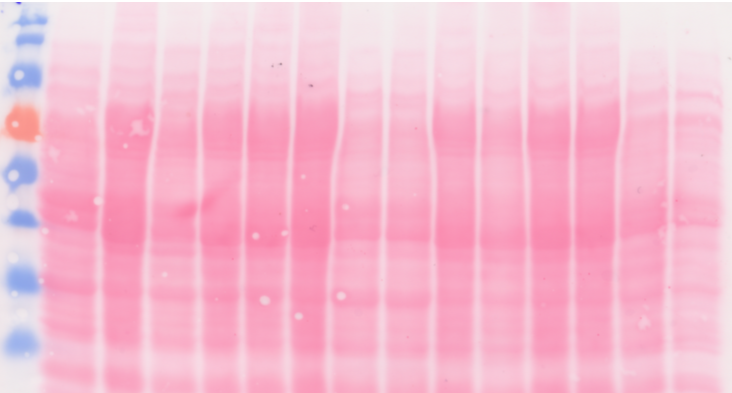

Supplement: Source data 2. [file elife-75143-data2.zip › Fig.6A +methanol - Ponceau S_Raw.tif]

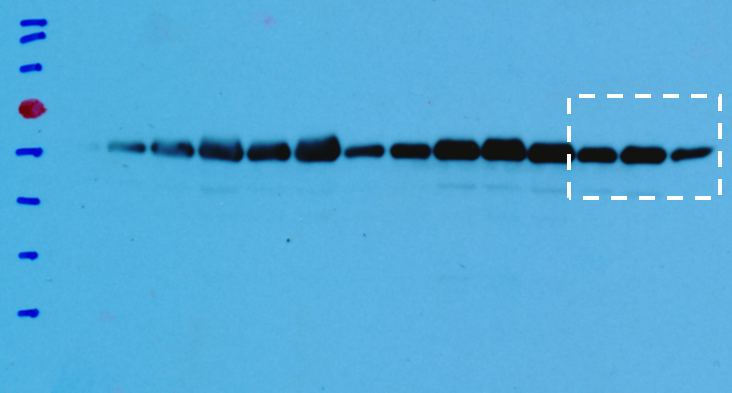

Supplement: Source data 2. [file elife-75143-data2.zip › Fig.6A +oleate - Pex3_Annotated.tif]

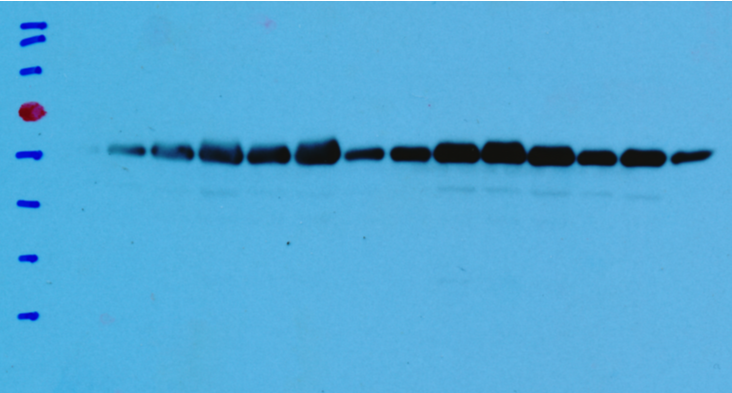

Supplement: Source data 2. [file elife-75143-data2.zip › Fig.6A +oleate - Pex3_Raw.tif]

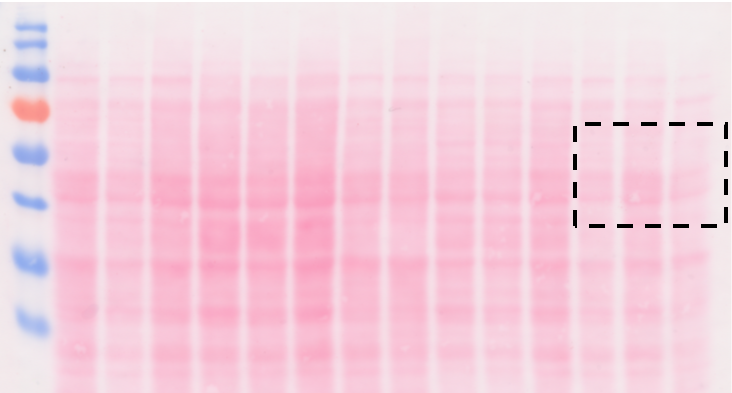

Supplement: Source data 2. [file elife-75143-data2.zip › Fig.6A +oleate - Ponceau S_Annotated.tif]

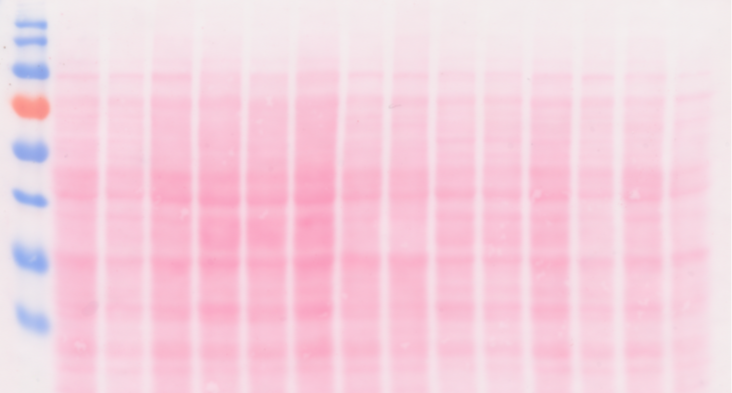

Supplement: Source data 2. [file elife-75143-data2.zip › Fig.6A +oleate - Ponceau S_Raw.tif]

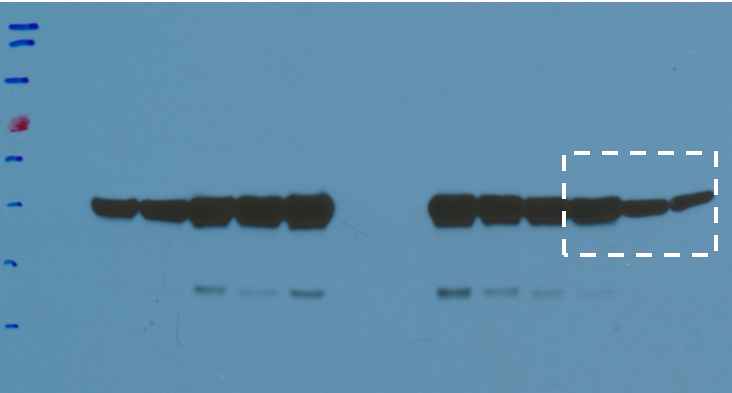

Supplement: Source data 2. [file elife-75143-data2.zip › Fig.6A +oleate - Pot1_Annotated.tif]

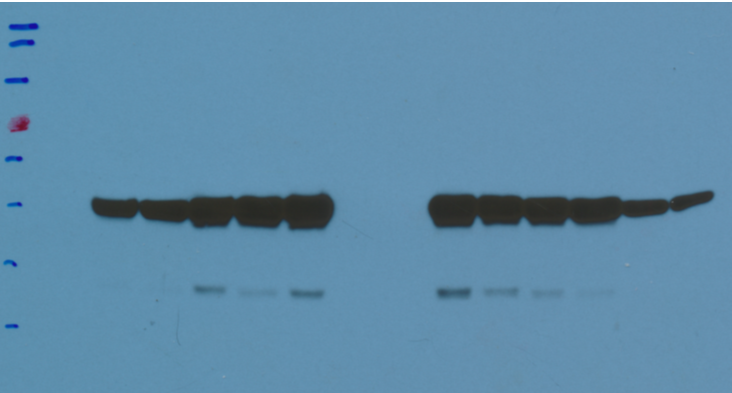

Supplement: Source data 2. [file elife-75143-data2.zip › Fig.6A +oleate -Pot1_Raw.tif]

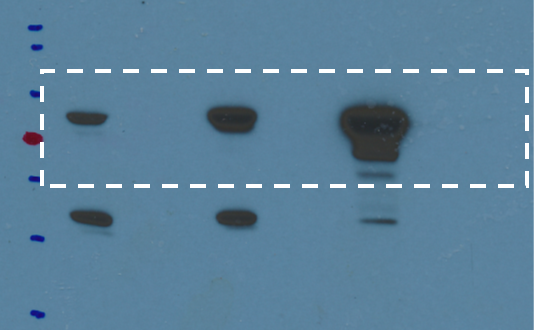

Supplement: Source data 2. [file elife-75143-data2.zip › Fig.6B +methanol -Aox1_Annotated.tif]

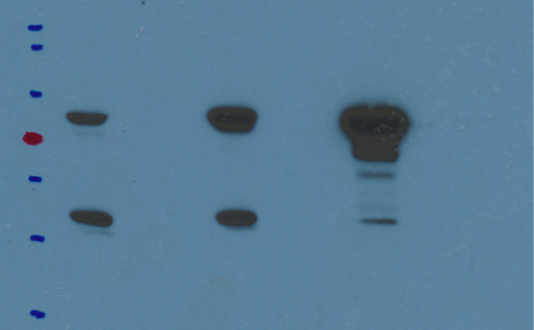

Supplement: Source data 2. [file elife-75143-data2.zip › Fig.6B +methanol -Aox1_Raw.tif]

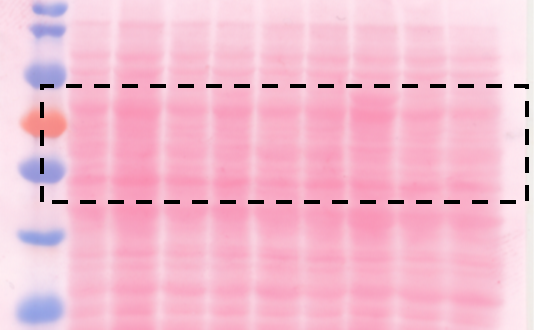

Supplement: Source data 2. [file elife-75143-data2.zip › Fig.6B +methanol -Ponceau S_Annotated.tif]

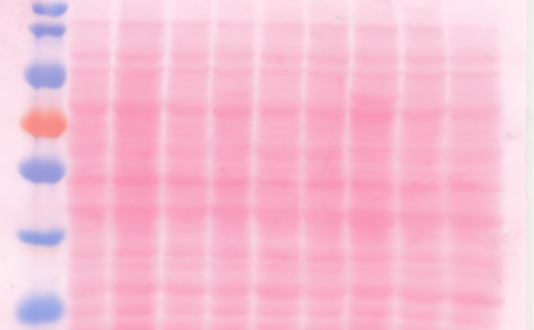

Supplement: Source data 2. [file elife-75143-data2.zip › Fig.6B +methanol -Ponceau S_Raw.tif]

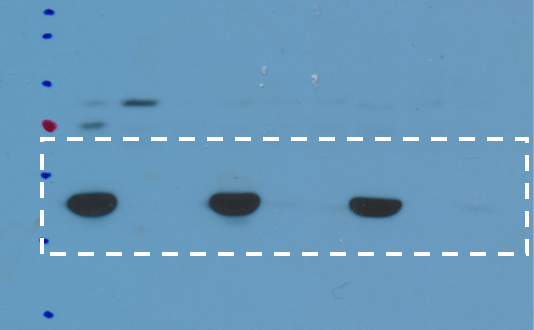

Supplement: Source data 2. [file elife-75143-data2.zip › Fig.6B +methanol -Pot1_Annotated.tif]

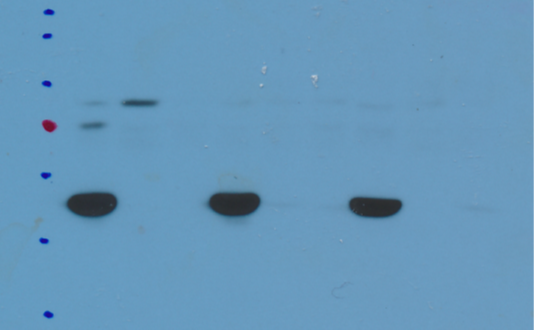

Supplement: Source data 2. [file elife-75143-data2.zip › Fig.6B +methanol -Pot1_Raw.tif]

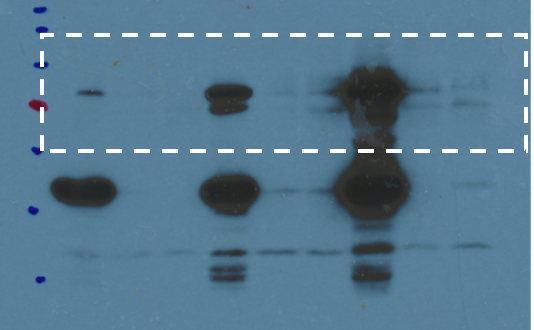

Supplement: Source data 2. [file elife-75143-data2.zip › Fig.6B +oleate -Aox1_Annotated.tif]

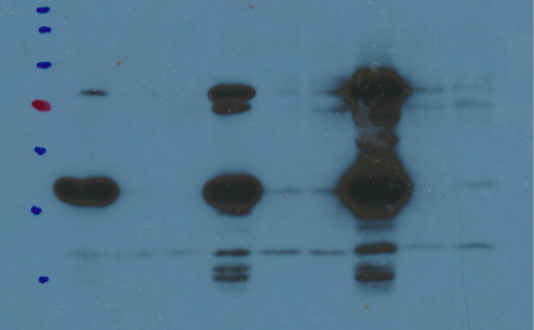

Supplement: Source data 2. [file elife-75143-data2.zip › Fig.6B +oleate -Aox1_Raw.tif]

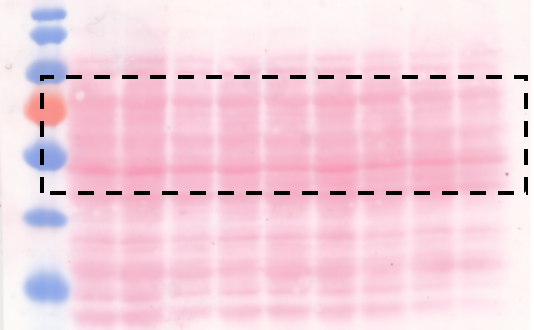

Supplement: Source data 2. [file elife-75143-data2.zip › Fig.6B +oleate -Ponceau S_Annotated.tif]

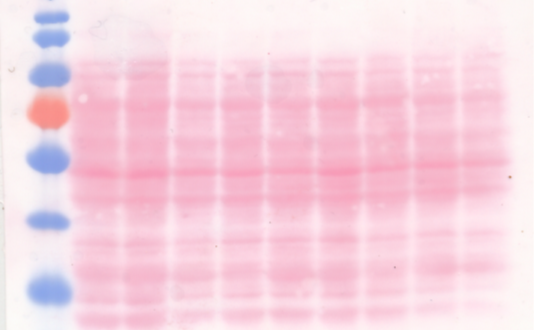

Supplement: Source data 2. [file elife-75143-data2.zip › Fig.6B +oleate -Ponceau S_Raw.tif]

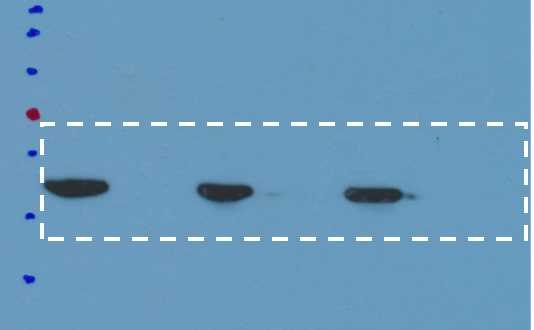

Supplement: Source data 2. [file elife-75143-data2.zip › Fig.6B +oleate -Pot1_Annotated.tif]

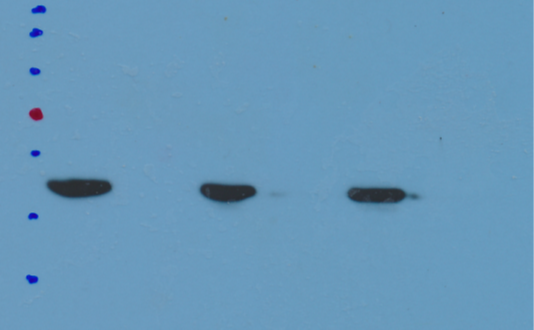

Supplement: Source data 2. [file elife-75143-data2.zip › Fig.6B +oleate -Pot1_Raw.tif]

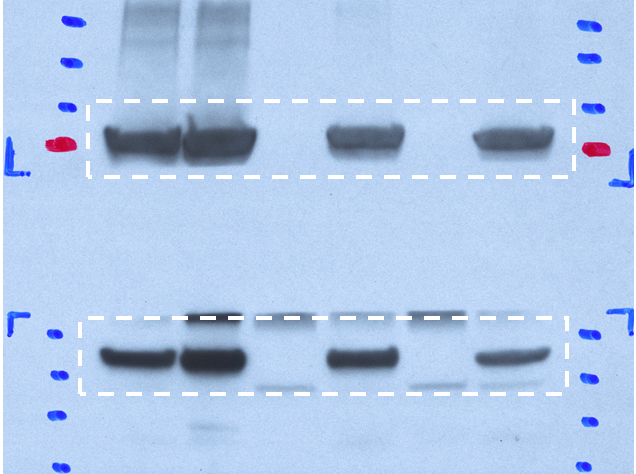

Supplement: Source data 2. [file elife-75143-data2.zip › Fig.7A +methanol - Aox1&Pot1_Annotated.tif]

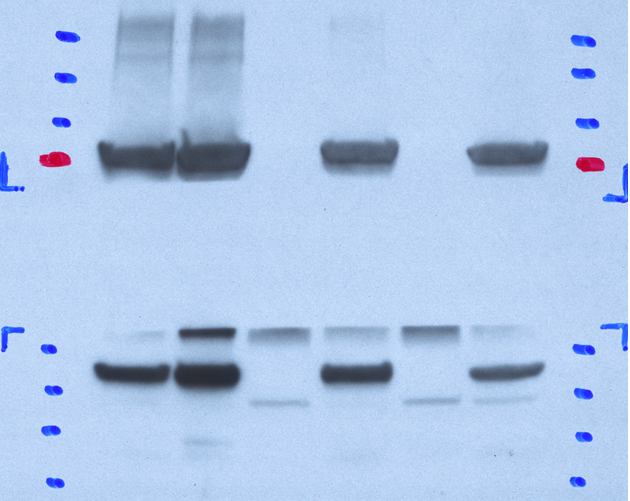

Supplement: Source data 2. [file elife-75143-data2.zip › Fig.7A +methanol - Aox1&Pot1_Raw.tif]

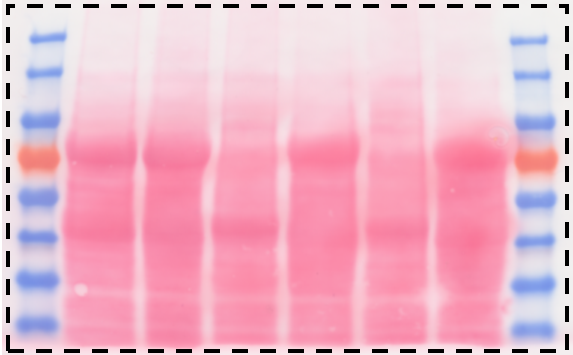

Supplement: Source data 2. [file elife-75143-data2.zip › Fig.7A +methanol - Ponceau S_Annotated.tif]

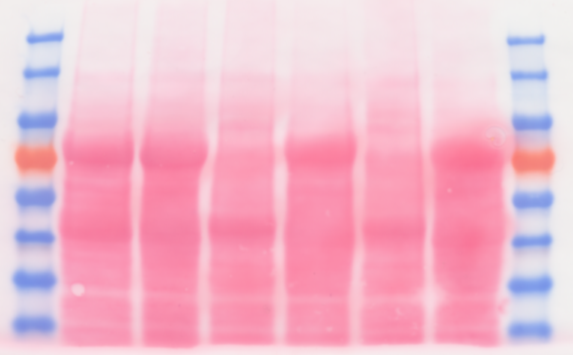

Supplement: Source data 2. [file elife-75143-data2.zip › Fig.7A +methanol - Ponceau S_Raw.tif]

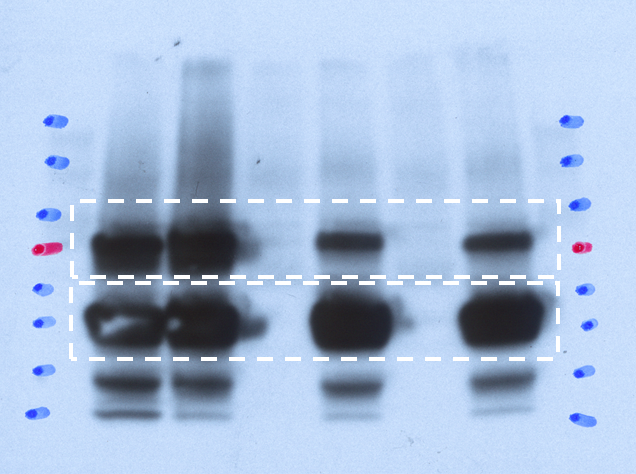

Supplement: Source data 2. [file elife-75143-data2.zip › Fig.7A +oleate - Aox1&Pot1_Annotated.tif]

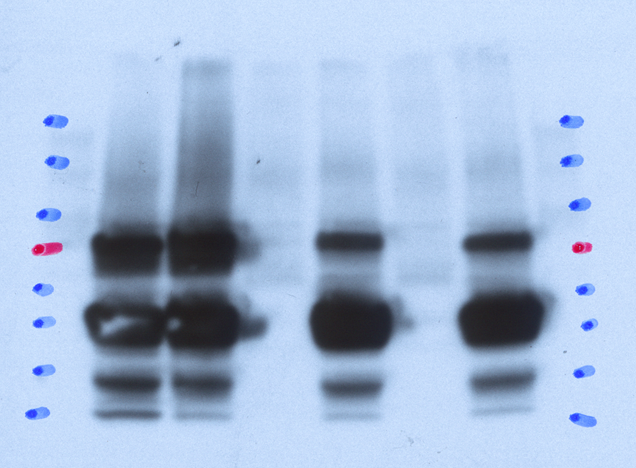

Supplement: Source data 2. [file elife-75143-data2.zip › Fig.7A +oleate - Aox1&Pot1_Raw.tif]

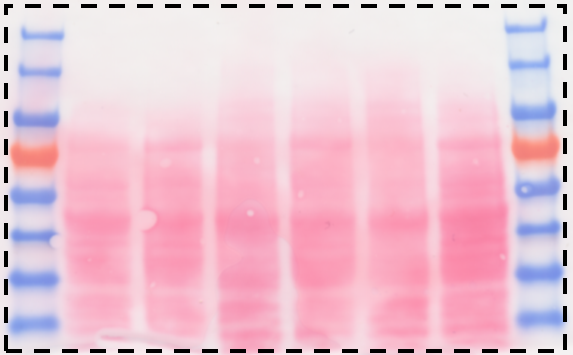

Supplement: Source data 2. [file elife-75143-data2.zip › Fig.7A +oleate - Ponceau S_Annotated.tif]

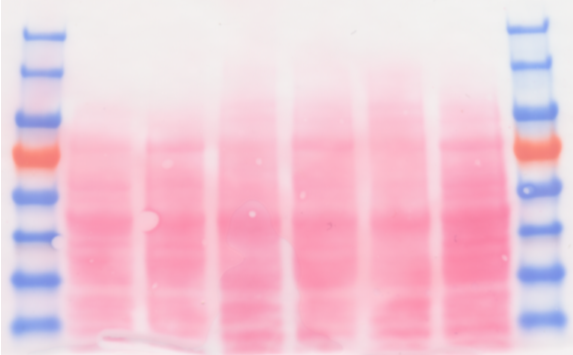

Supplement: Source data 2. [file elife-75143-data2.zip › Fig.7A +oleate - Ponceau S_Raw.tif]
