# Supplementary figures and images for: OXPHOS deficiencies affect peroxisome proliferation by downregulating genes controlled by the SNF1 signaling pathway (part 1 of 2)
Source: eLife. 2022 Apr 25;11:e75143. doi: 10.7554/eLife.75143 (PMC9094750; doi:10.7554/eLife.75143)

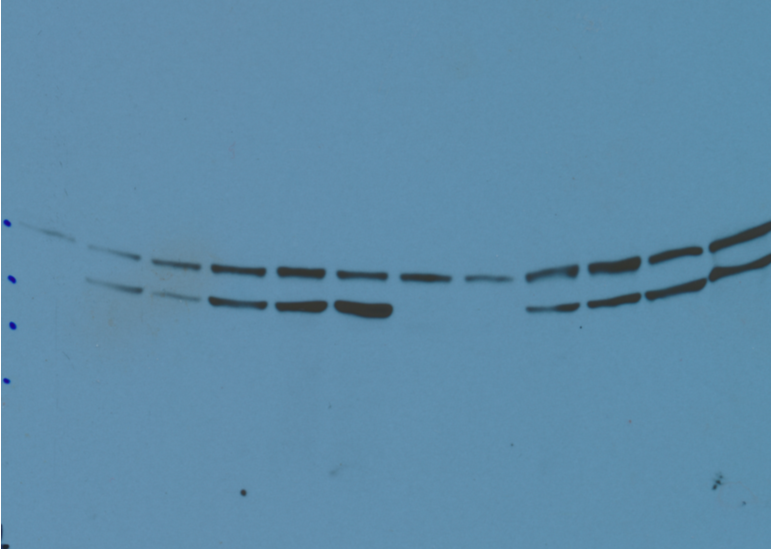

Supplement: Source data 1. [file elife-75143-data1.zip › Fig. 4C +methanol - Pot1_Raw.tif]

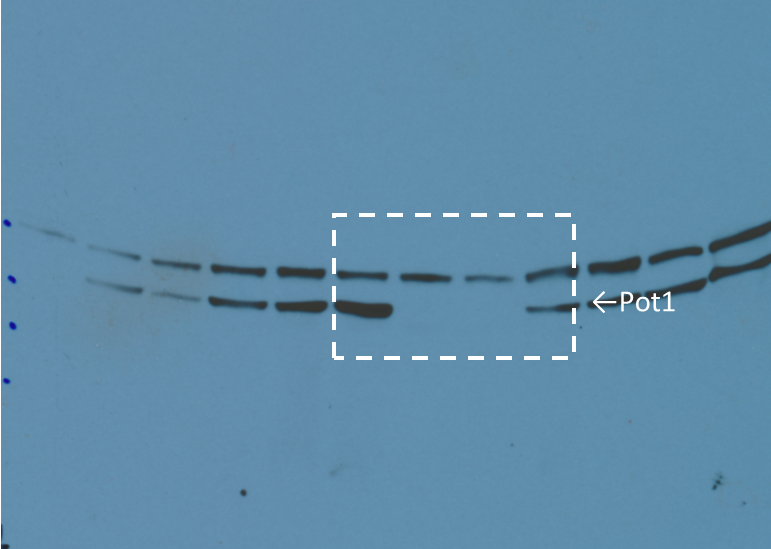

Supplement: Source data 1. [file elife-75143-data1.zip › Fig. 4C +methanol - Pot1_Annotated.tif]

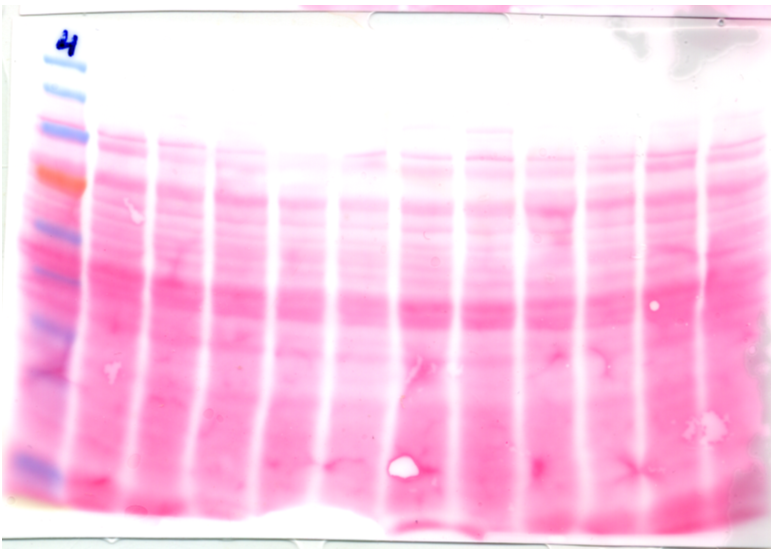

Supplement: Source data 1. [file elife-75143-data1.zip › Fig. 4C +methanol - Ponceau S_Raw.tif]

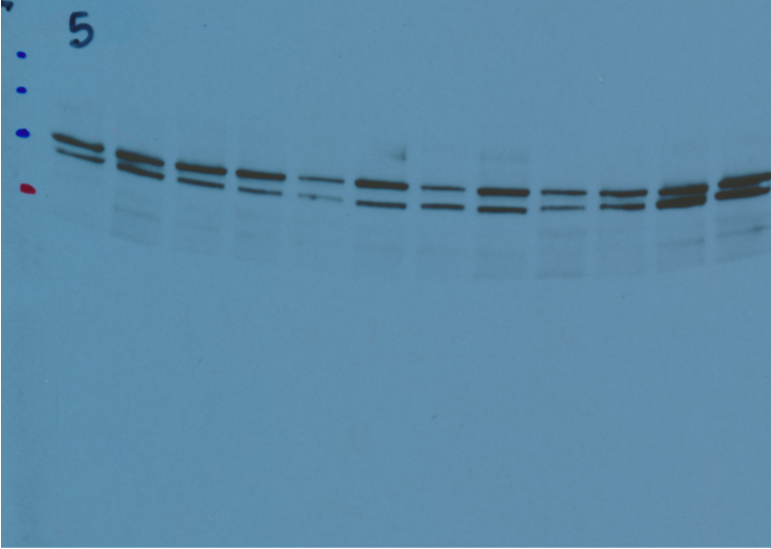

Supplement: Source data 1. [file elife-75143-data1.zip › Fig. 4C +oleate - Aox1_Raw.tif]

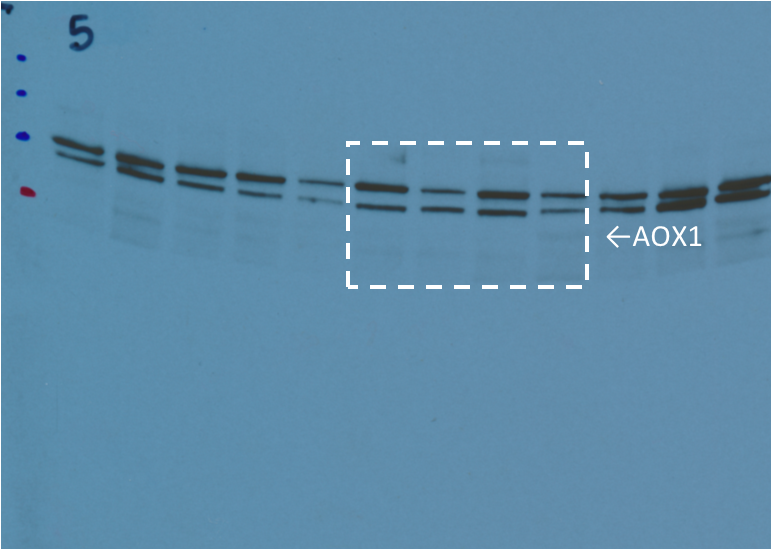

Supplement: Source data 1. [file elife-75143-data1.zip › Fig. 4C +oleate - Aox1_Annotated.tif]

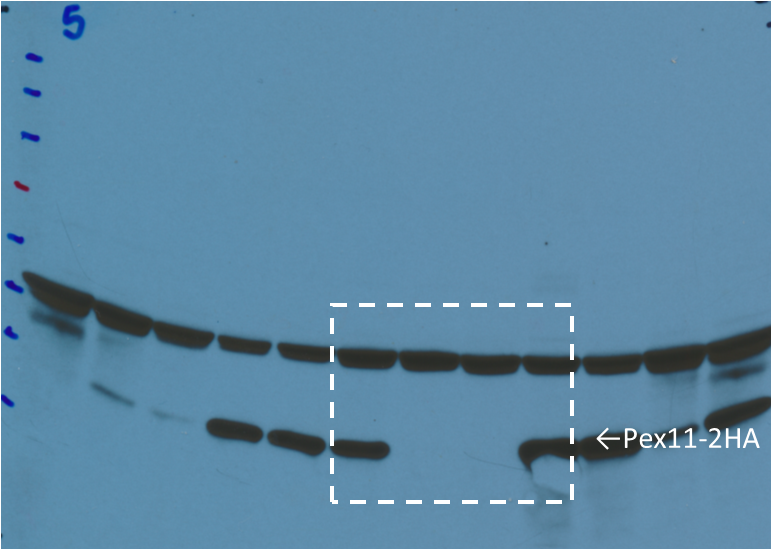

Supplement: Source data 1. [file elife-75143-data1.zip › Fig. 4C +oleate - HA_Annotated.tif]

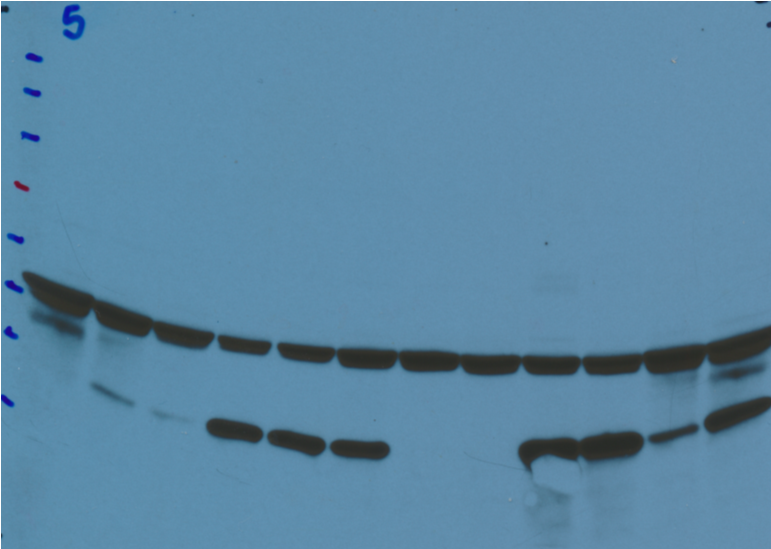

Supplement: Source data 1. [file elife-75143-data1.zip › Fig. 4C +oleate - HA_Raw.tif]

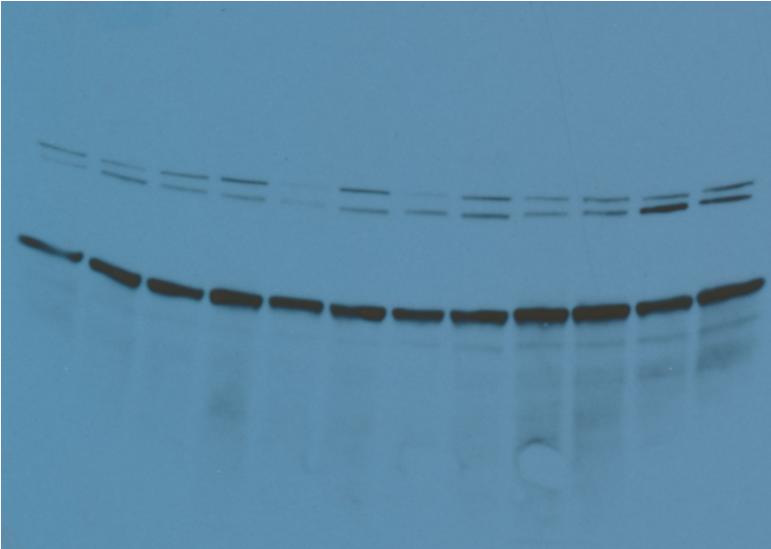

Supplement: Source data 1. [file elife-75143-data1.zip › Fig. 4C +oleate - Pex3_Raw.tif]

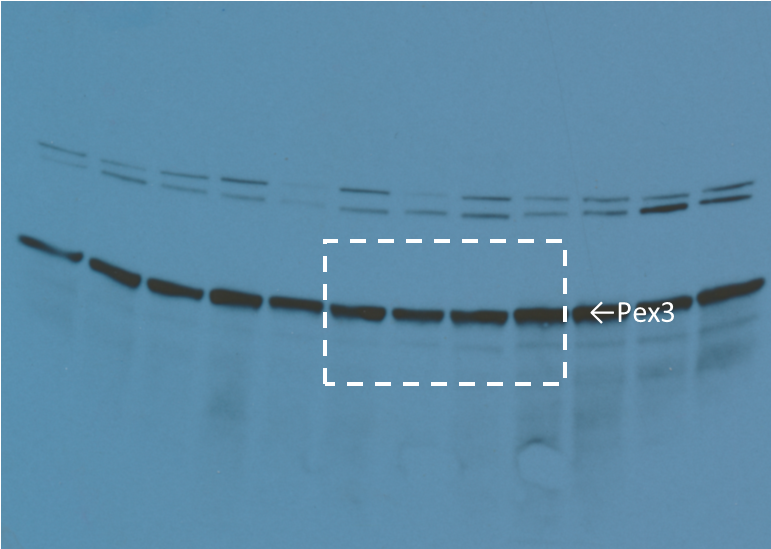

Supplement: Source data 1. [file elife-75143-data1.zip › Fig. 4C +oleate - Pex3_Annotated.tif]

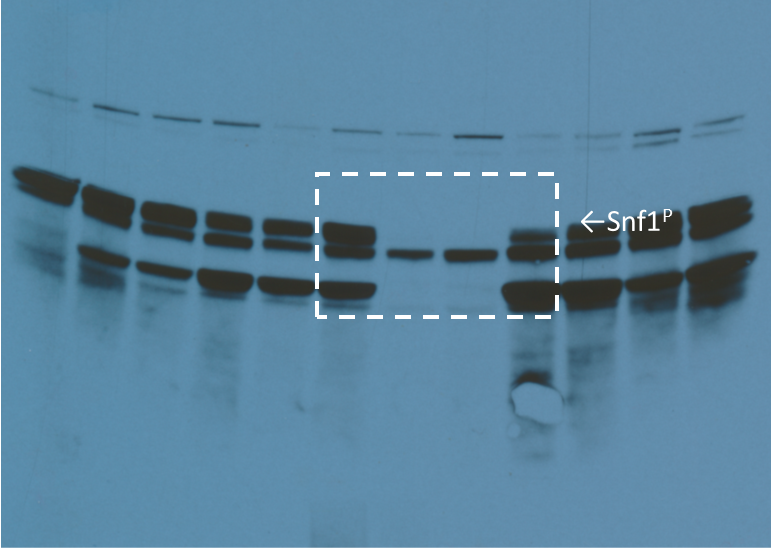

Supplement: Source data 1. [file elife-75143-data1.zip › Fig. 4C +oleate - Phospho Snf1_Annotated.tif]

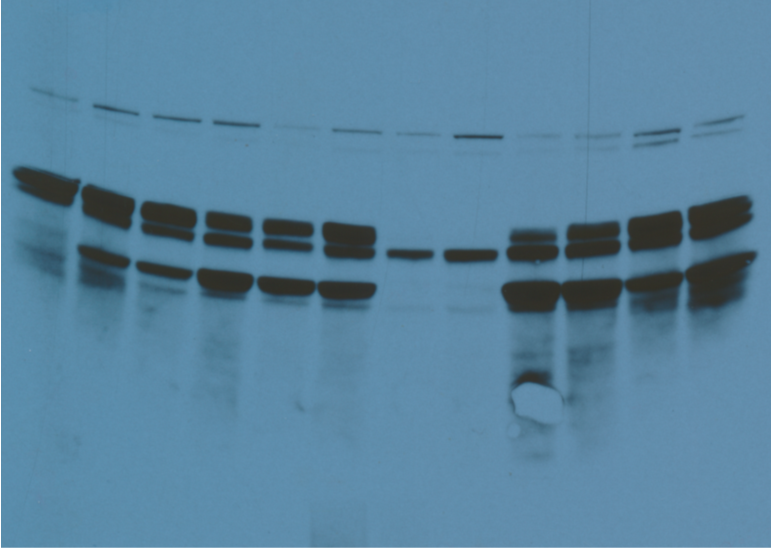

Supplement: Source data 1. [file elife-75143-data1.zip › Fig. 4C +oleate - Phospho Snf1_Raw.tif]

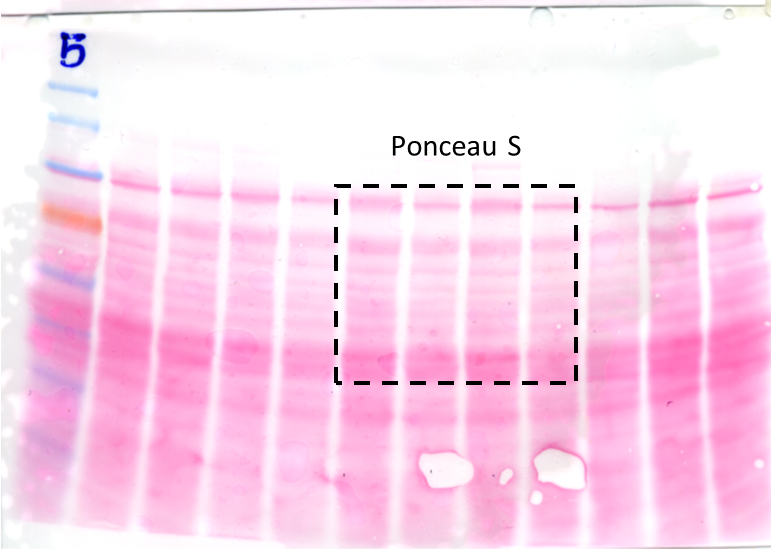

Supplement: Source data 1. [file elife-75143-data1.zip › Fig. 4C +oleate - Ponceau S_Annotated.tif]

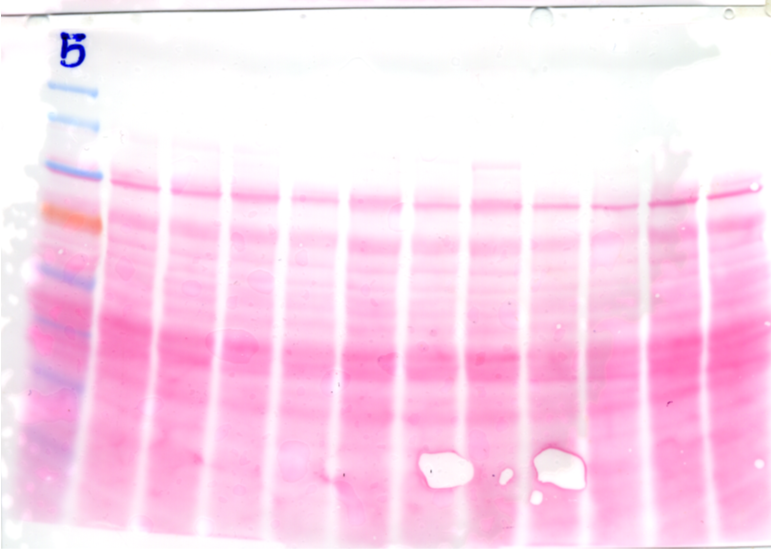

Supplement: Source data 1. [file elife-75143-data1.zip › Fig. 4C +oleate - Ponceau S_Raw.tif]

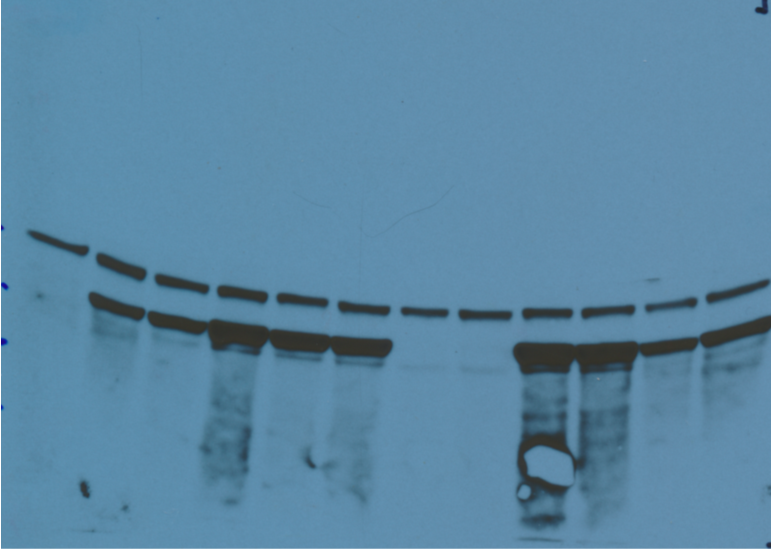

Supplement: Source data 1. [file elife-75143-data1.zip › Fig. 4C +oleate - Pot1_Raw.tif]

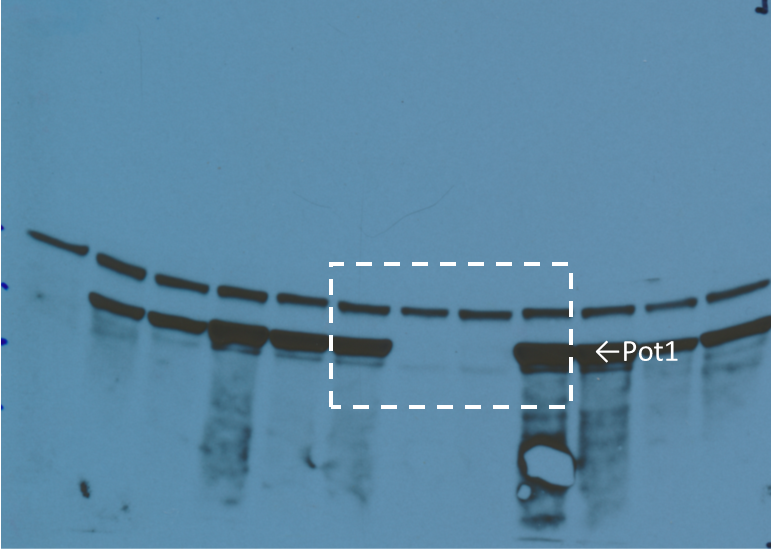

Supplement: Source data 1. [file elife-75143-data1.zip › Fig. 4C +oleate - Pot1_Annotated.tif]

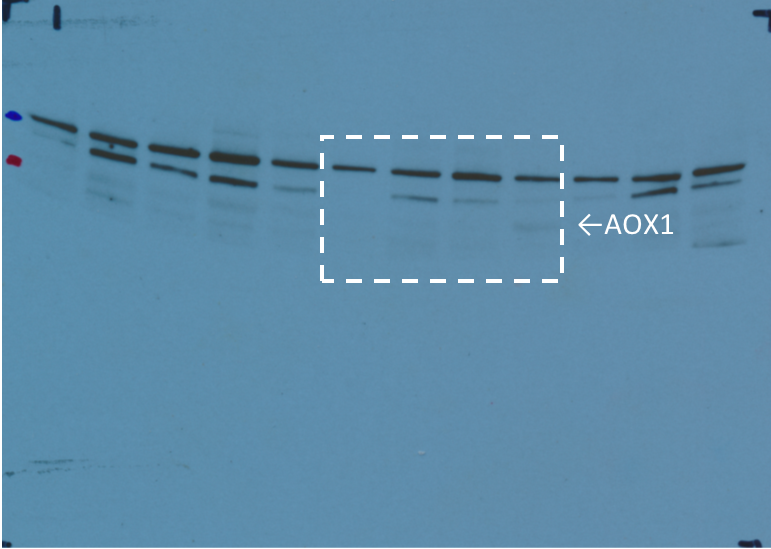

Supplement: Source data 1. [file elife-75143-data1.zip › Fig. 4C -glucose - Aox1_Annotated.tif]

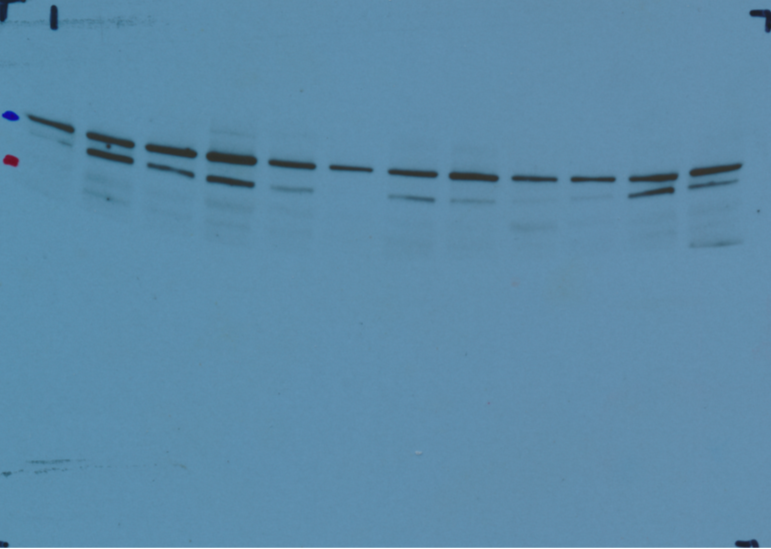

Supplement: Source data 1. [file elife-75143-data1.zip › Fig. 4C -glucose - Aox1_Raw.tif]

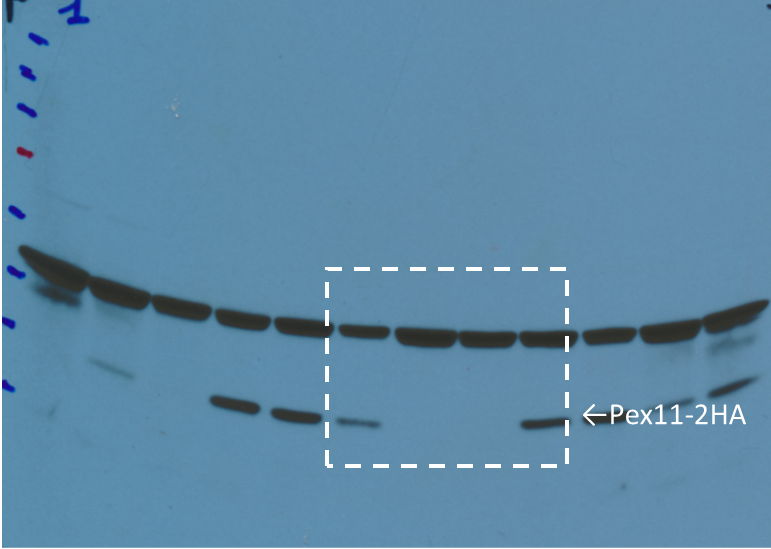

Supplement: Source data 1. [file elife-75143-data1.zip › Fig. 4C -glucose - HA_Annotated.tif]

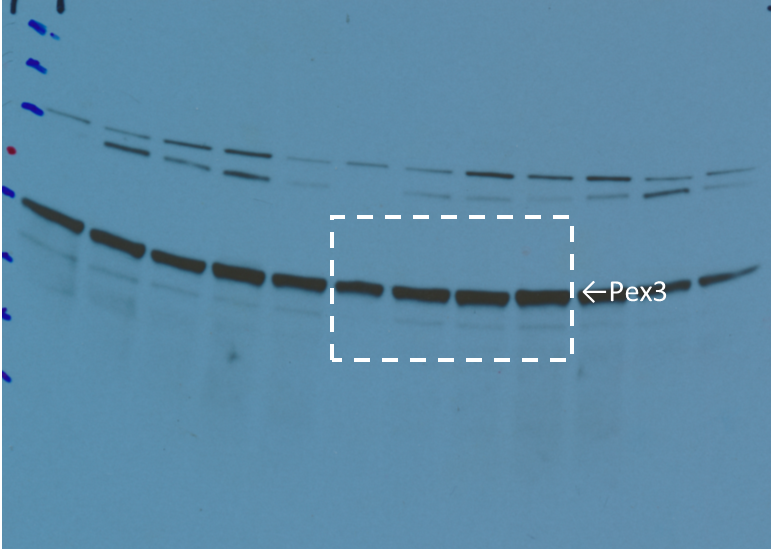

Supplement: Source data 1. [file elife-75143-data1.zip › Fig. 4C -glucose - Pex3_Annotated.tif]

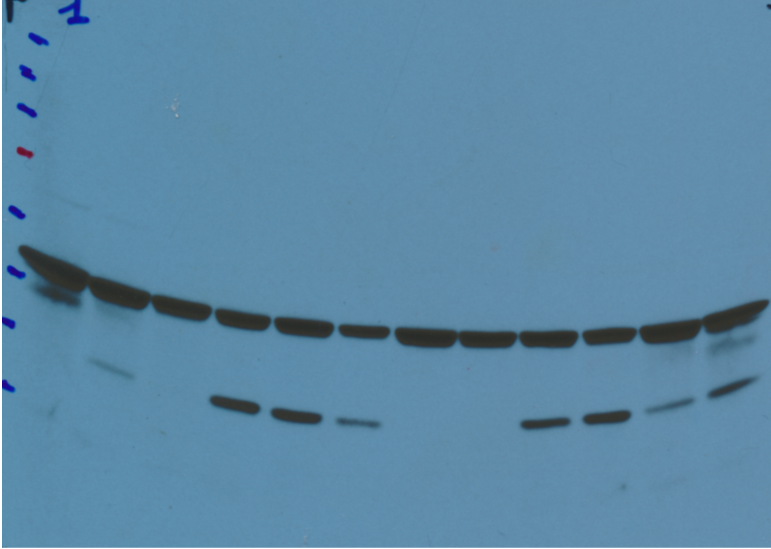

Supplement: Source data 1. [file elife-75143-data1.zip › Fig. 4C -glucose - HA_Raw.tif]

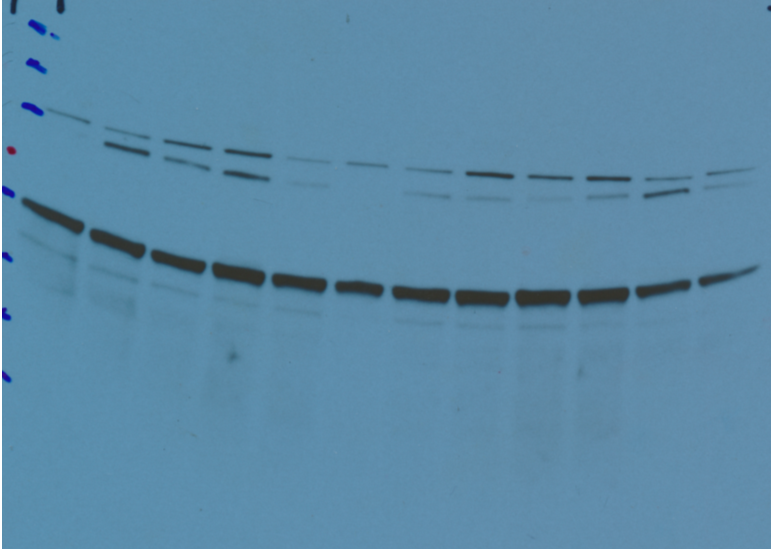

Supplement: Source data 1. [file elife-75143-data1.zip › Fig. 4C -glucose - Pex3_Raw.tif]

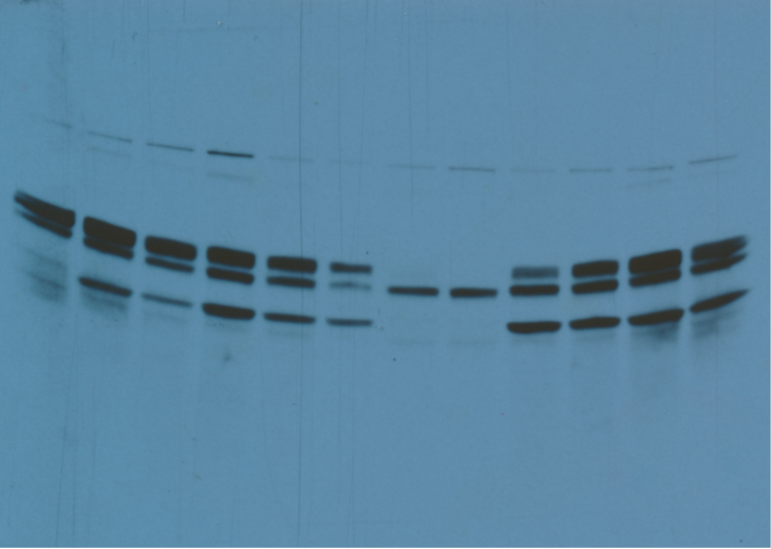

Supplement: Source data 1. [file elife-75143-data1.zip › Fig. 4C -glucose - Phospho Snf1_Raw.tif]

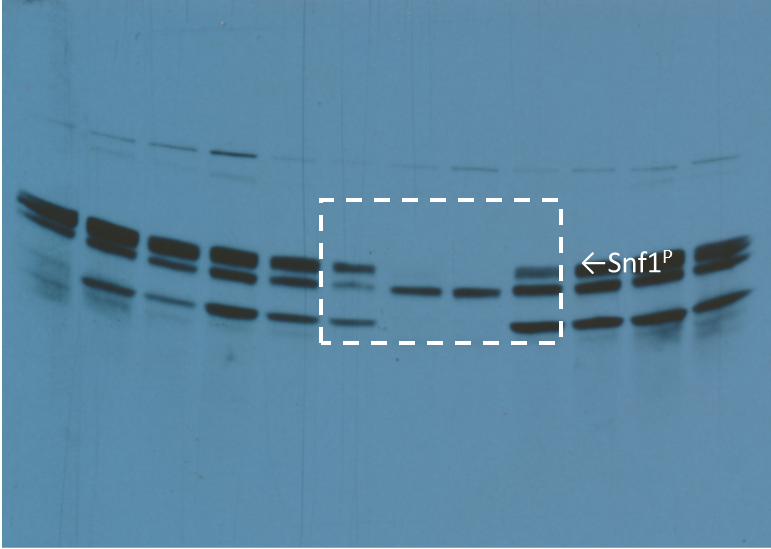

Supplement: Source data 1. [file elife-75143-data1.zip › Fig. 4C -glucose - Phospho Snf1_Annotated.tif]

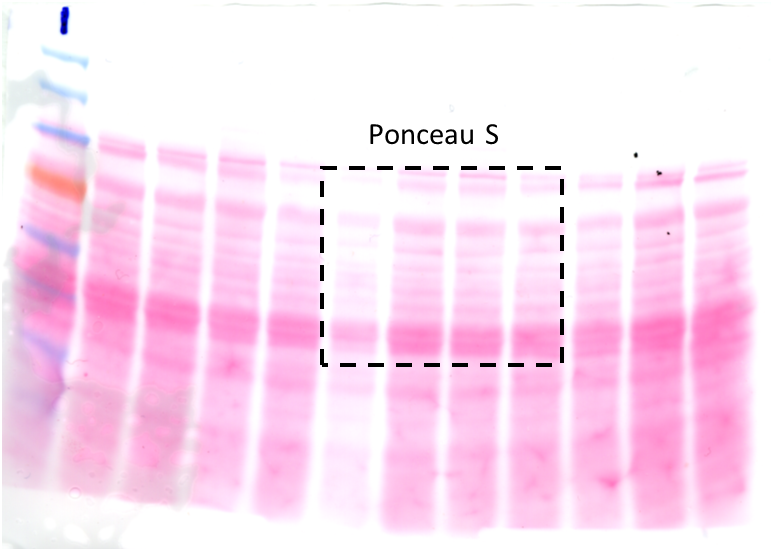

Supplement: Source data 1. [file elife-75143-data1.zip › Fig. 4C -glucose - Ponceau S_Annotated.tif]

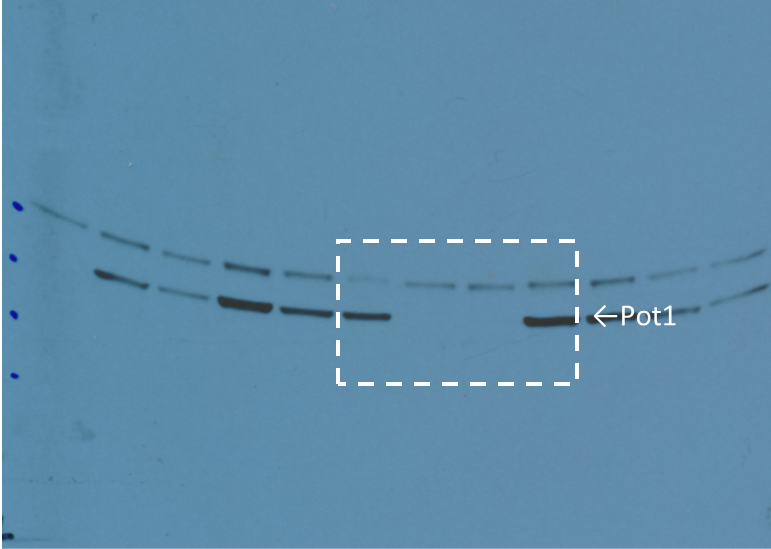

Supplement: Source data 1. [file elife-75143-data1.zip › Fig. 4C -glucose - Pot1_Annotated.tif]

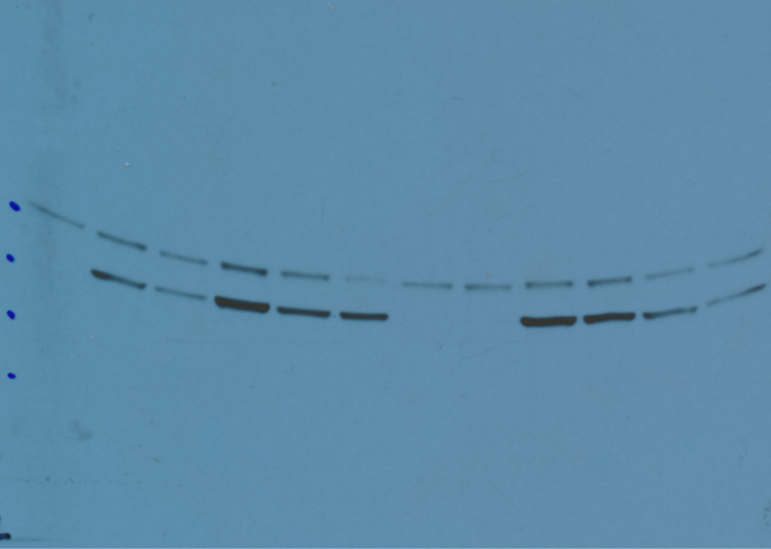

Supplement: Source data 1. [file elife-75143-data1.zip › Fig. 4C -glucose - Pot1_Raw.tif]

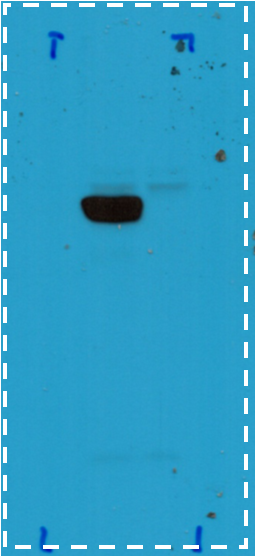

Supplement: Source data 1. [file elife-75143-data1.zip › Fig. 3_Figure supplement 1 -Aox1_Annotated.tif]

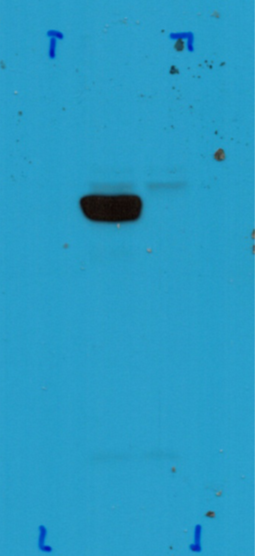

Supplement: Source data 1. [file elife-75143-data1.zip › Fig. 3_Figure supplement 1 -Aox1_Raw.tif]

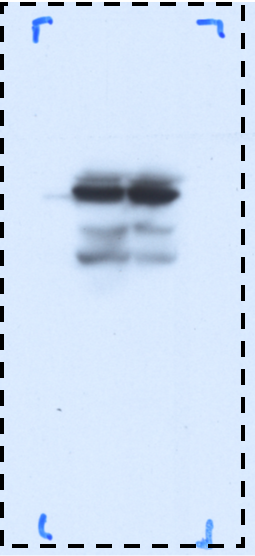

Supplement: Source data 1. [file elife-75143-data1.zip › Fig. 3_Figure supplement 1 -GFP_Annotated.tif]

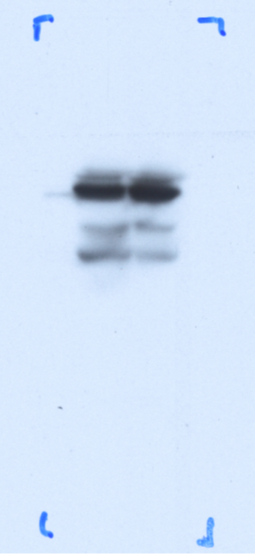

Supplement: Source data 1. [file elife-75143-data1.zip › Fig. 3_Figure supplement 1 -GFP_Raw.tif]

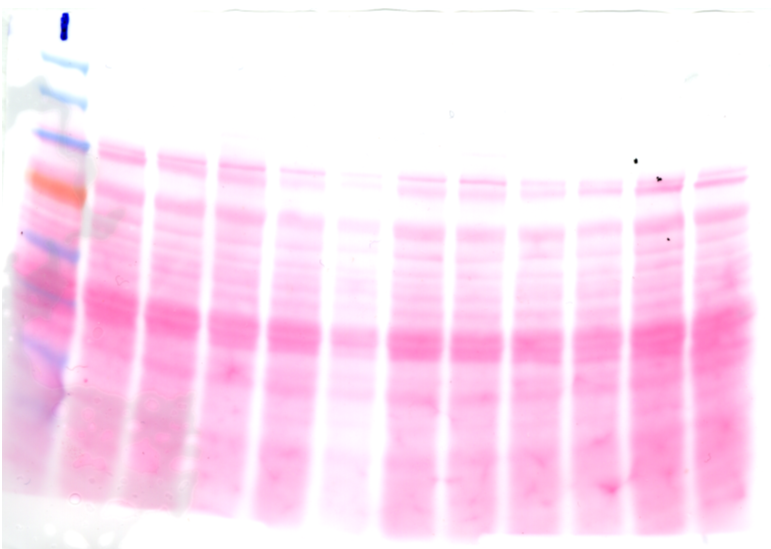

Supplement: Source data 1. [file elife-75143-data1.zip › Fig. 4C -glucose - Ponceau S_Raw.tif]

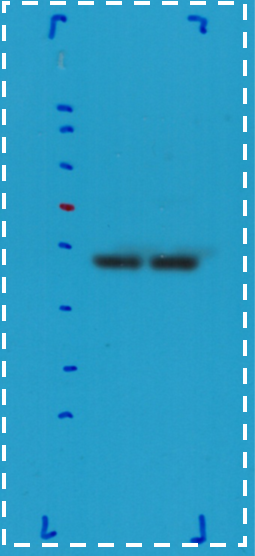

Supplement: Source data 1. [file elife-75143-data1.zip › Fig. 3_Figure supplement 1 -Phospho Snf1_Annotated.tif]

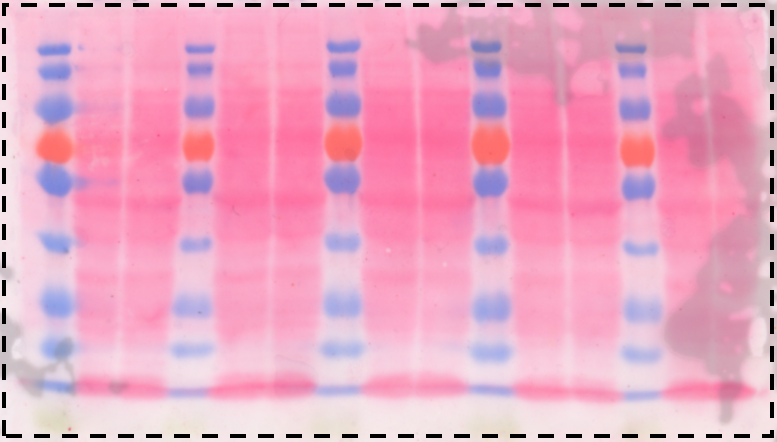

Supplement: Source data 1. [file elife-75143-data1.zip › Fig. 3_Figure supplement 1 -Ponceau S_Annotated.tif]

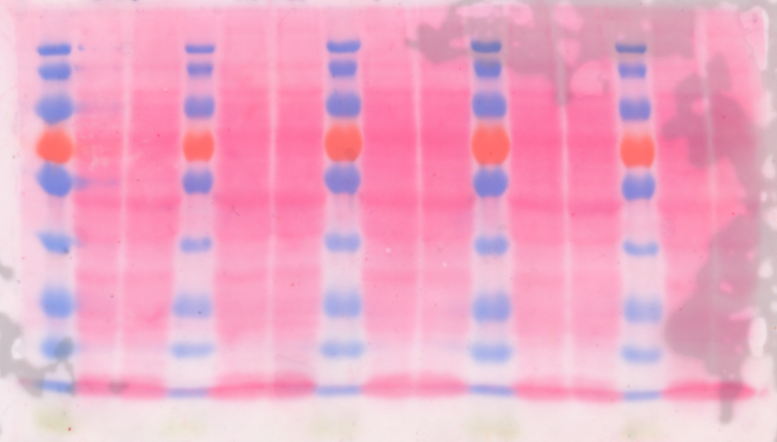

Supplement: Source data 1. [file elife-75143-data1.zip › Fig. 3_Figure supplement 1 -Ponceau S_Raw.tif]

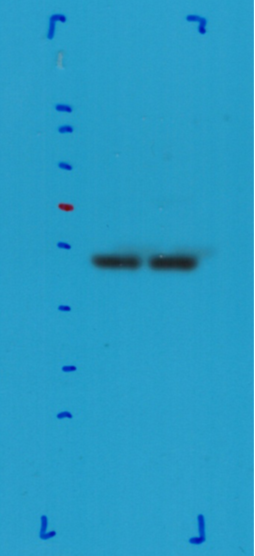

Supplement: Source data 1. [file elife-75143-data1.zip › Fig. 3_Figure supplement 1 -Phospho Snf1_Raw.tif]

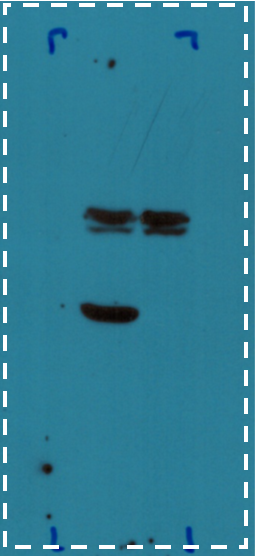

Supplement: Source data 1. [file elife-75143-data1.zip › Fig. 3_Figure supplement 1 -Pot1_Annotated.tif]

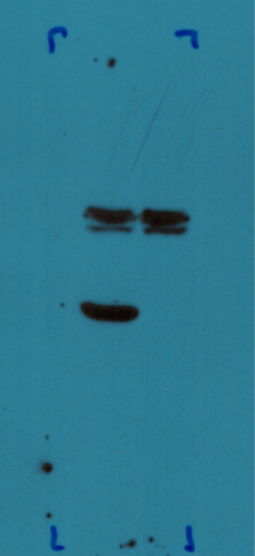

Supplement: Source data 1. [file elife-75143-data1.zip › Fig. 3_Figure supplement 1 -Pot1_Raw.tif]

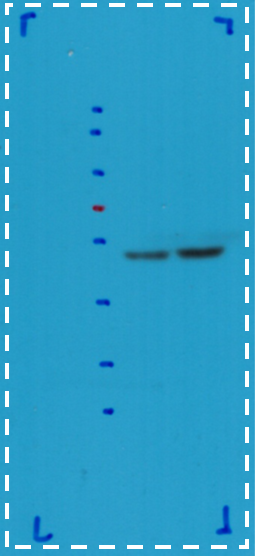

Supplement: Source data 1. [file elife-75143-data1.zip › Fig. 3_Figure supplement 1 -Snf1 total_Annotated.tif]

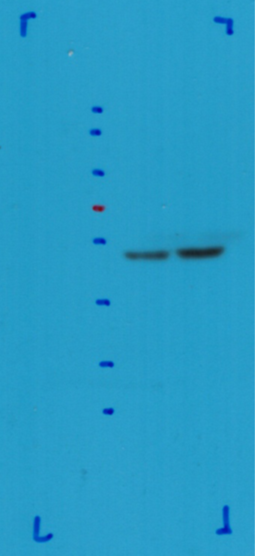

Supplement: Source data 1. [file elife-75143-data1.zip › Fig. 3_Figure supplement 1 -Snf1 total_Raw.tif]

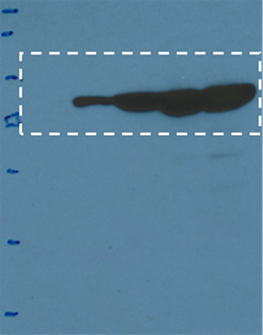

Supplement: Source data 1. [file elife-75143-data1.zip › Fig. 4_Figure supplement 1A +methanol -Aox1_Annotated.tif]

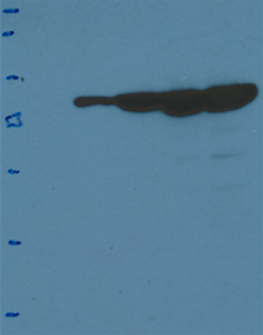

Supplement: Source data 1. [file elife-75143-data1.zip › Fig. 4_Figure supplement 1A +methanol -Aox1_Raw.tif]

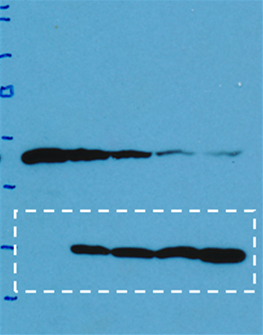

Supplement: Source data 1. [file elife-75143-data1.zip › Fig. 4_Figure supplement 1A +methanol -HA_Annotated.tif]

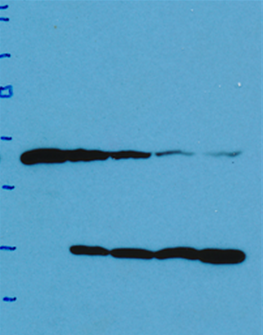

Supplement: Source data 1. [file elife-75143-data1.zip › Fig. 4_Figure supplement 1A +methanol -HA_Raw.tif]

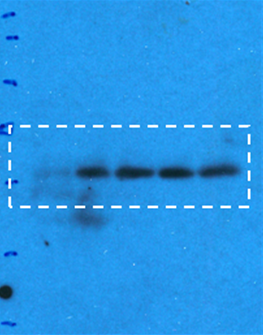

Supplement: Source data 1. [file elife-75143-data1.zip › Fig. 4_Figure supplement 1A +methanol -Pex2_Annotated.tif]

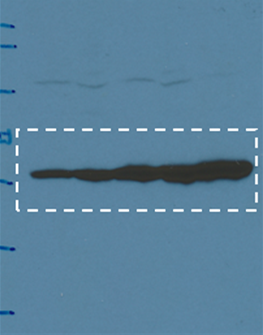

Supplement: Source data 1. [file elife-75143-data1.zip › Fig. 4_Figure supplement 1A +methanol -Pex3_Annotated.tif]

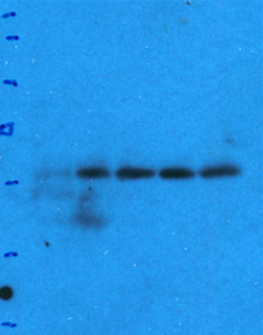

Supplement: Source data 1. [file elife-75143-data1.zip › Fig. 4_Figure supplement 1A +methanol -Pex2_Raw.tif]

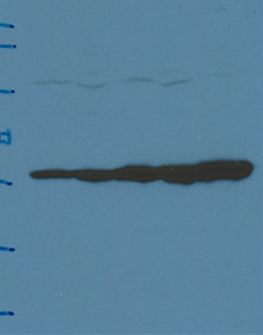

Supplement: Source data 1. [file elife-75143-data1.zip › Fig. 4_Figure supplement 1A +methanol -Pex3_Raw.tif]

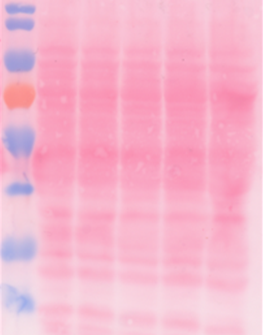

Supplement: Source data 1. [file elife-75143-data1.zip › Fig. 4_Figure supplement 1A +methanol -Ponceau S for HA_Raw.tif]

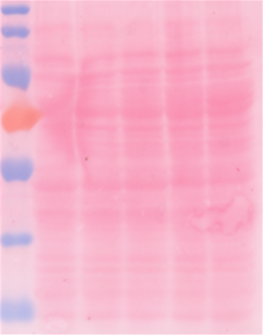

Supplement: Source data 1. [file elife-75143-data1.zip › Fig. 4_Figure supplement 1A +methanol -Ponceau S for Aox1_Raw.tif]

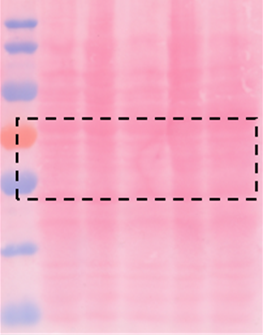

Supplement: Source data 1. [file elife-75143-data1.zip › Fig. 4_Figure supplement 1A +methanol -Ponceau S for Pex3_Annotated.tif]

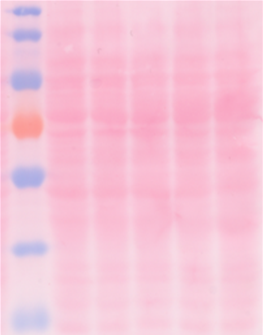

Supplement: Source data 1. [file elife-75143-data1.zip › Fig. 4_Figure supplement 1A +methanol -Ponceau S for Pex2_Raw.tif]

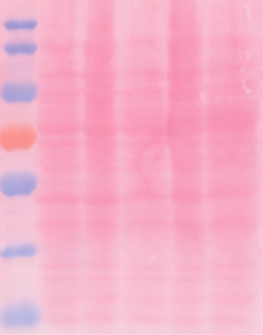

Supplement: Source data 1. [file elife-75143-data1.zip › Fig. 4_Figure supplement 1A +methanol -Ponceau S for Pex3_Raw.tif]

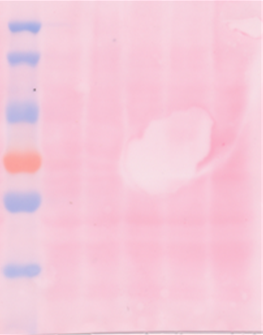

Supplement: Source data 1. [file elife-75143-data1.zip › Fig. 4_Figure supplement 1A +methanol -Ponceau S for Pot1_Raw.tif]

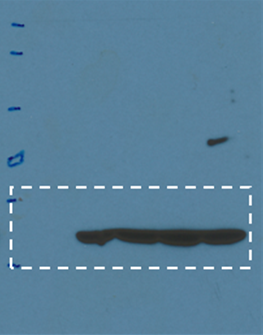

Supplement: Source data 1. [file elife-75143-data1.zip › Fig. 4_Figure supplement 1A +methanol -Pot1_Annotated.tif]

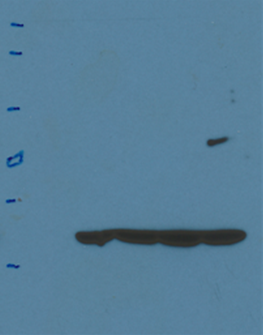

Supplement: Source data 1. [file elife-75143-data1.zip › Fig. 4_Figure supplement 1A +methanol -Pot1_Raw.tif]

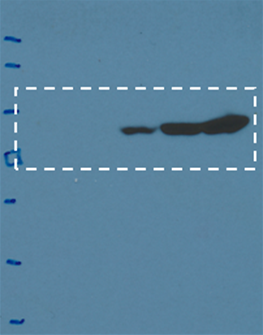

Supplement: Source data 1. [file elife-75143-data1.zip › Fig. 4_Figure supplement 1A +oleate -Aox1_Annotated.tif]

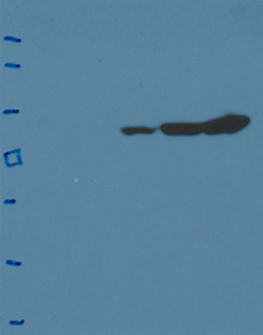

Supplement: Source data 1. [file elife-75143-data1.zip › Fig. 4_Figure supplement 1A +oleate -Aox1_Raw.tif]

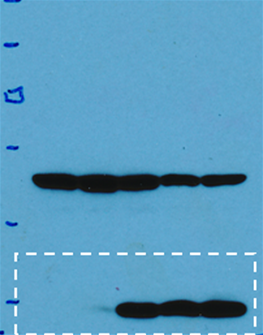

Supplement: Source data 1. [file elife-75143-data1.zip › Fig. 4_Figure supplement 1A +oleate -HA_Annotated.tif]

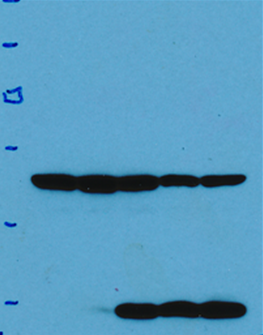

Supplement: Source data 1. [file elife-75143-data1.zip › Fig. 4_Figure supplement 1A +oleate -HA_Raw.tif]

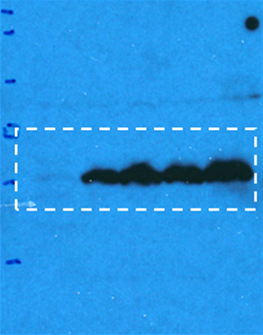

Supplement: Source data 1. [file elife-75143-data1.zip › Fig. 4_Figure supplement 1A +oleate -Pex2_Annotated.tif]

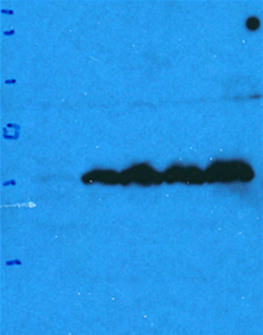

Supplement: Source data 1. [file elife-75143-data1.zip › Fig. 4_Figure supplement 1A +oleate -Pex2_Raw.tif]

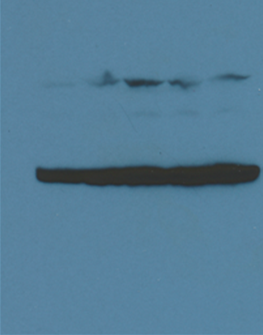

Supplement: Source data 1. [file elife-75143-data1.zip › Fig. 4_Figure supplement 1A +oleate -Pex3_Raw.tif]

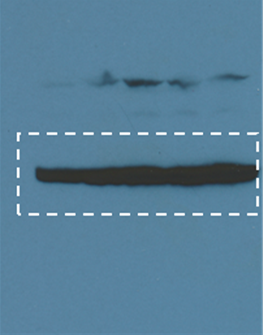

Supplement: Source data 1. [file elife-75143-data1.zip › Fig. 4_Figure supplement 1A +oleate -Pex3_Annotated.tif]

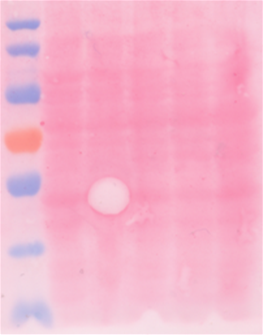

Supplement: Source data 1. [file elife-75143-data1.zip › Fig. 4_Figure supplement 1A +oleate -Ponceau S for Aox1_Raw.tif]

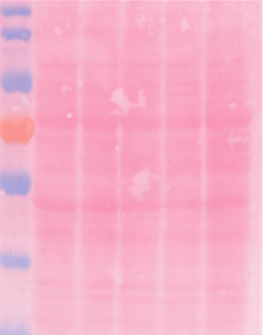

Supplement: Source data 1. [file elife-75143-data1.zip › Fig. 4_Figure supplement 1A +oleate -Ponceau S for Pex2_Raw.tif]

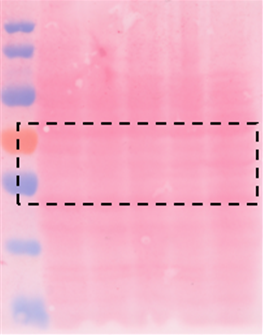

Supplement: Source data 1. [file elife-75143-data1.zip › Fig. 4_Figure supplement 1A +oleate -Ponceau S for Pex3_Annotated.tif]

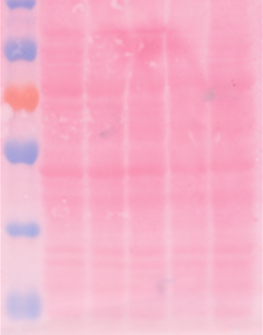

Supplement: Source data 1. [file elife-75143-data1.zip › Fig. 4_Figure supplement 1A +oleate -Ponceau S for HA_Raw.tif]

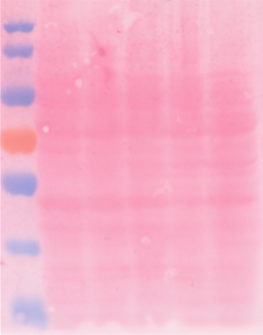

Supplement: Source data 1. [file elife-75143-data1.zip › Fig. 4_Figure supplement 1A +oleate -Ponceau S for Pex3_Raw.tif]

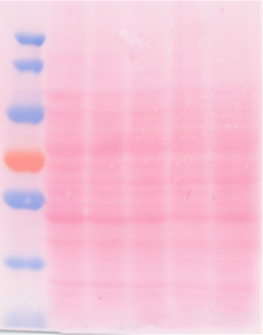

Supplement: Source data 1. [file elife-75143-data1.zip › Fig. 4_Figure supplement 1A +oleate -Ponceau S for Pot1_Raw.tif]

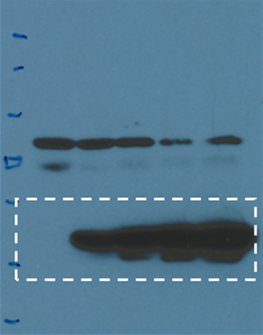

Supplement: Source data 1. [file elife-75143-data1.zip › Fig. 4_Figure supplement 1A +oleate -Pot1_Annotated.tif]

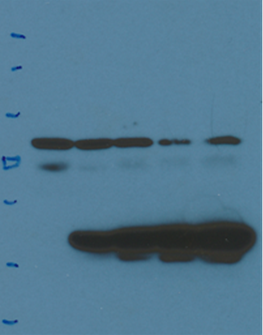

Supplement: Source data 1. [file elife-75143-data1.zip › Fig. 4_Figure supplement 1A +oleate -Pot1_Raw.tif]

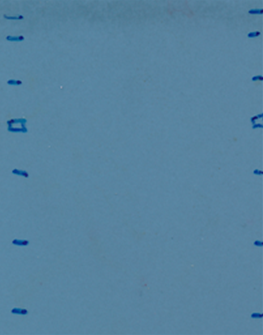

Supplement: Source data 1. [file elife-75143-data1.zip › Fig. 4_Figure supplement 1A -glucose -Aox1_Raw.tif]

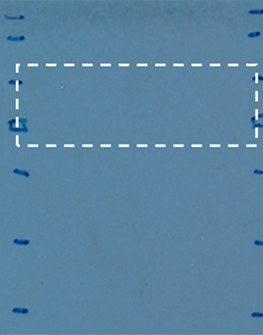

Supplement: Source data 1. [file elife-75143-data1.zip › Fig. 4_Figure supplement 1A -glucose -Aox1_Annotated.tif]

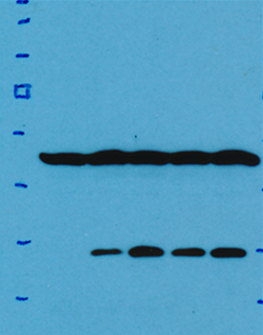

Supplement: Source data 1. [file elife-75143-data1.zip › Fig. 4_Figure supplement 1A -glucose -HA_Raw.tif]

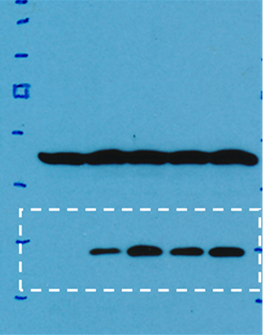

Supplement: Source data 1. [file elife-75143-data1.zip › Fig. 4_Figure supplement 1A -glucose -HA_Annotated.tif]

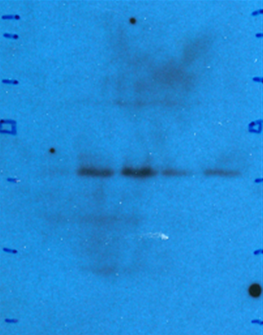

Supplement: Source data 1. [file elife-75143-data1.zip › Fig. 4_Figure supplement 1A -glucose -Pex2_Raw.tif]

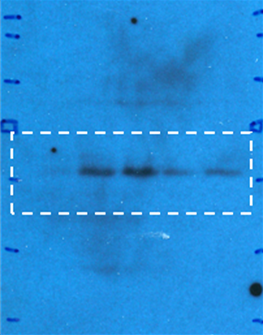

Supplement: Source data 1. [file elife-75143-data1.zip › Fig. 4_Figure supplement 1A -glucose -Pex2_Annotated.tif]

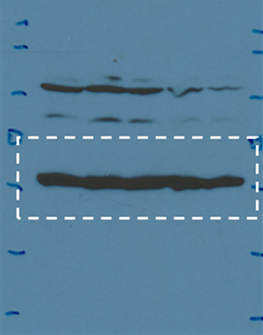

Supplement: Source data 1. [file elife-75143-data1.zip › Fig. 4_Figure supplement 1A -glucose -Pex3_Annotated.tif]

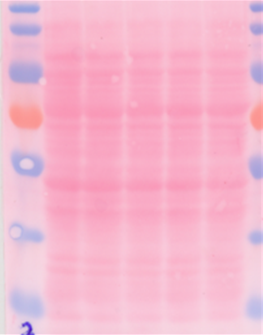

Supplement: Source data 1. [file elife-75143-data1.zip › Fig. 4_Figure supplement 1A -glucose -Ponceau S for Aox1_Raw.tif]

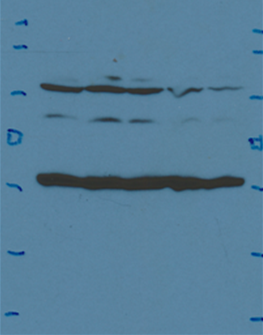

Supplement: Source data 1. [file elife-75143-data1.zip › Fig. 4_Figure supplement 1A -glucose -Pex3_Raw.tif]

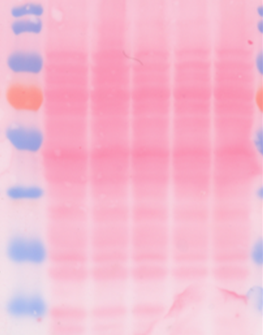

Supplement: Source data 1. [file elife-75143-data1.zip › Fig. 4_Figure supplement 1A -glucose -Ponceau S for HA_Raw.tif]

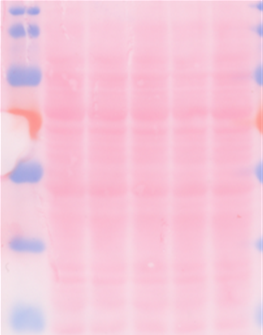

Supplement: Source data 1. [file elife-75143-data1.zip › Fig. 4_Figure supplement 1A -glucose -Ponceau S for Pex2_Raw.tif]

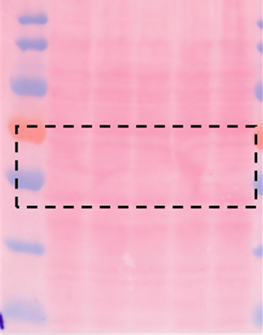

Supplement: Source data 1. [file elife-75143-data1.zip › Fig. 4_Figure supplement 1A -glucose -Ponceau S for Pex3_Annotated.tif]

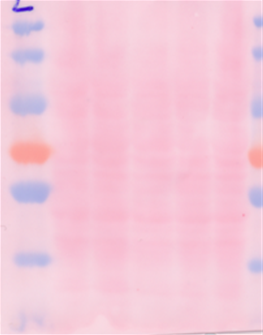

Supplement: Source data 1. [file elife-75143-data1.zip › Fig. 4_Figure supplement 1A -glucose -Ponceau S for Pot1_Raw.tif]

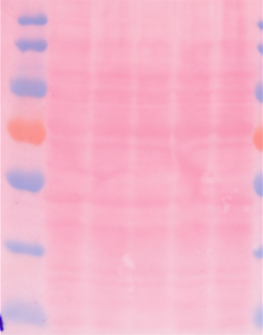

Supplement: Source data 1. [file elife-75143-data1.zip › Fig. 4_Figure supplement 1A -glucose -Ponceau S for Pex3_Raw.tif]

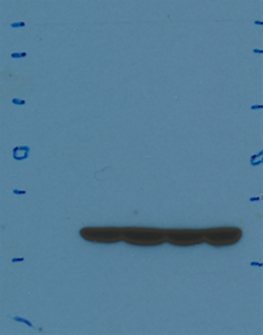

Supplement: Source data 1. [file elife-75143-data1.zip › Fig. 4_Figure supplement 1A -glucose -Pot1_Raw.tif]

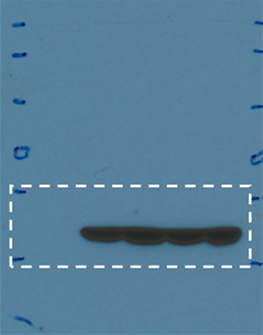

Supplement: Source data 1. [file elife-75143-data1.zip › Fig. 4_Figure supplement 1A -glucose -Pot1_Annotated.tif]

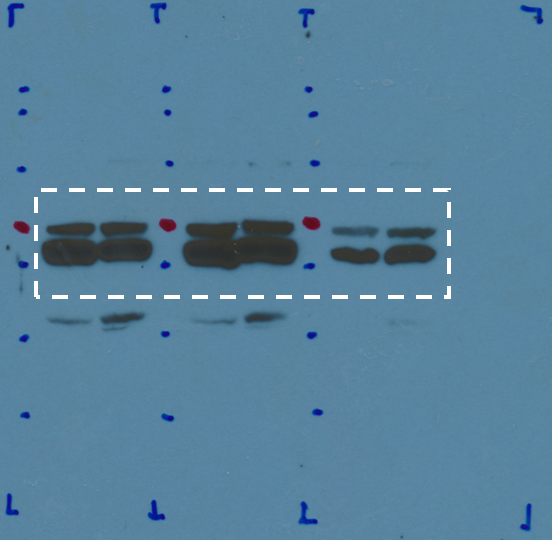

Supplement: Source data 1. [file elife-75143-data1.zip › Fig. 4_Figure supplement 1B - Snf1 total_Annotated.tif]

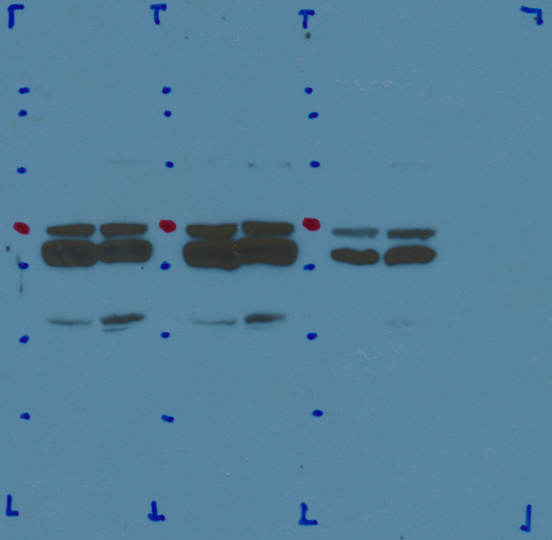

Supplement: Source data 1. [file elife-75143-data1.zip › Fig. 4_Figure supplement 1B - Snf1 total_Raw.tif]

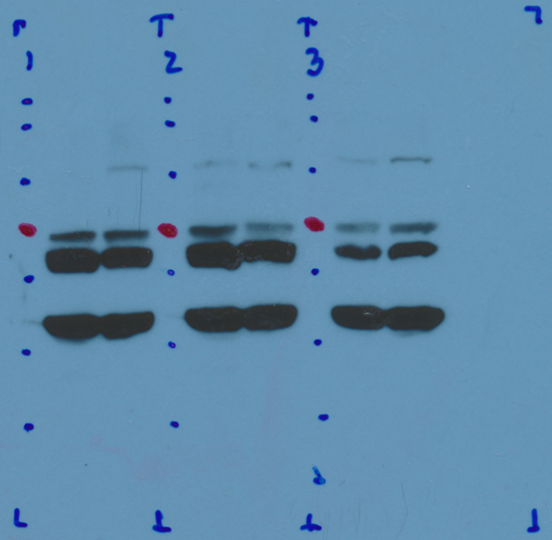

Supplement: Source data 1. [file elife-75143-data1.zip › Fig. 4_Figure supplement 1B - Actin_Raw.tif]

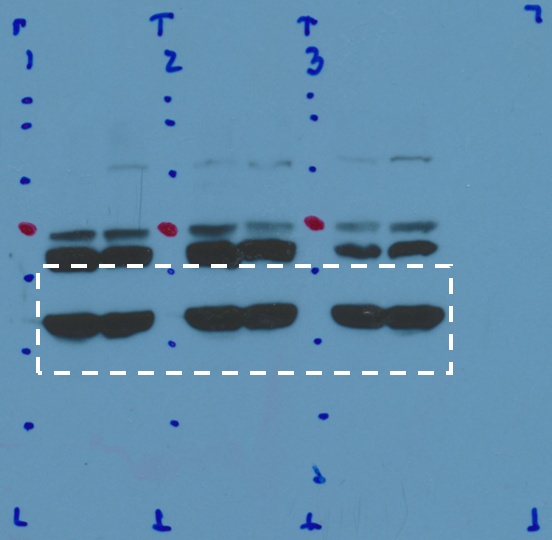

Supplement: Source data 1. [file elife-75143-data1.zip › Fig. 4_Figure supplement 1B- Actin_Annotated.tif]

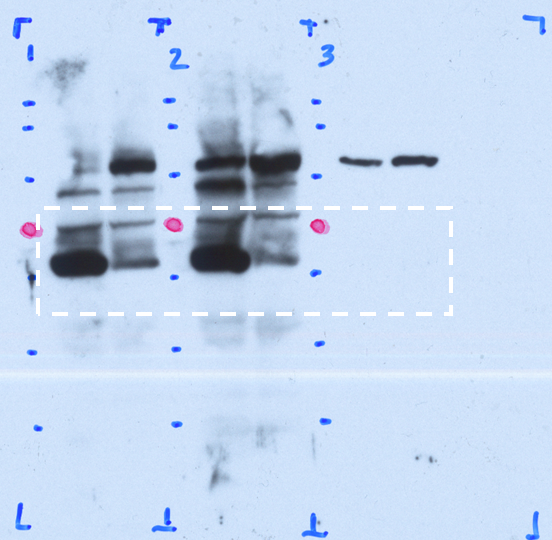

Supplement: Source data 1. [file elife-75143-data1.zip › Fig. 4_Figure supplement 1B -long expo - Phospho Snf1_Annotated.tif]

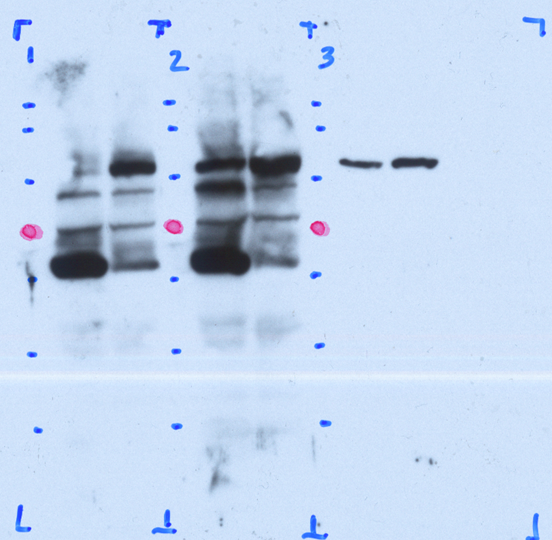

Supplement: Source data 1. [file elife-75143-data1.zip › Fig. 4_Figure supplement 1B -long expo - Phospho Snf1_Raw.tif]

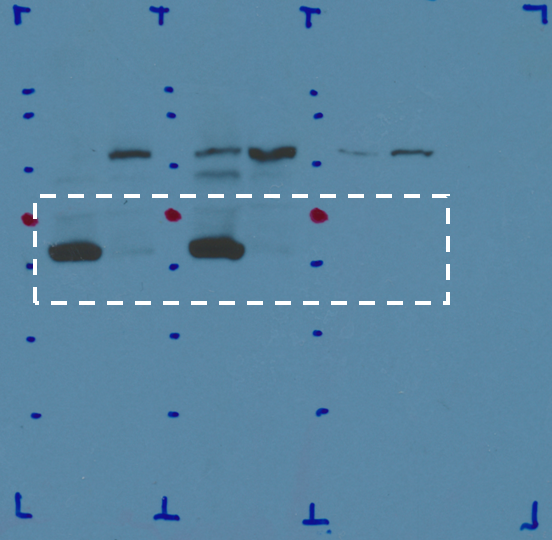

Supplement: Source data 1. [file elife-75143-data1.zip › Fig. 4_Figure supplement 1B -short expo - Phospho Snf1_Annotated.tif]

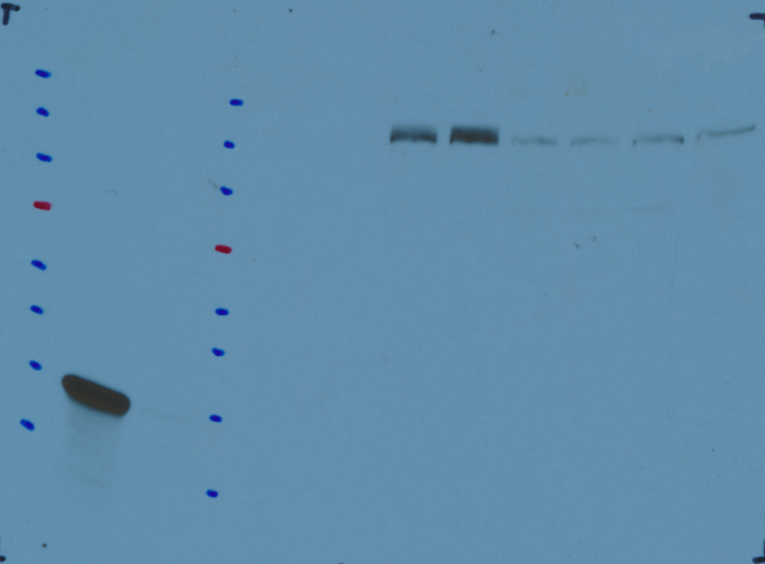

Supplement: Source data 1. [file elife-75143-data1.zip › Fig. 4A - HA_Raw.tif]

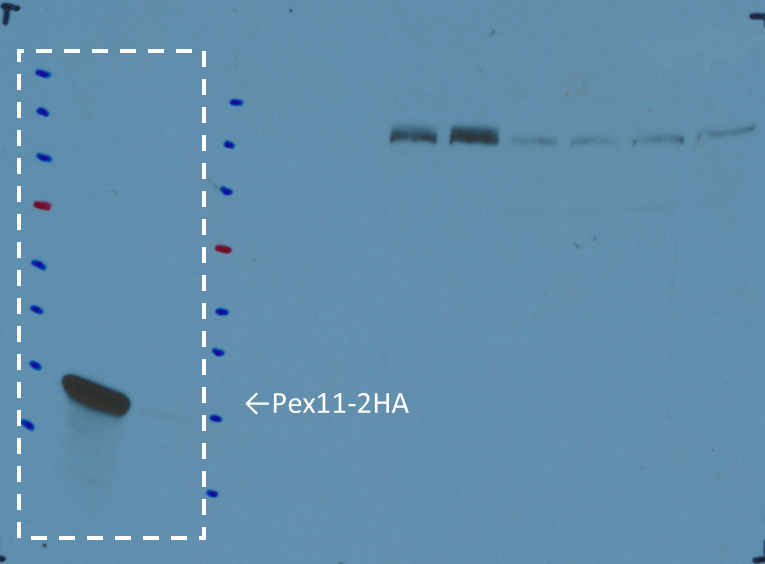

Supplement: Source data 1. [file elife-75143-data1.zip › Fig. 4A - HA_Annotated.tif]

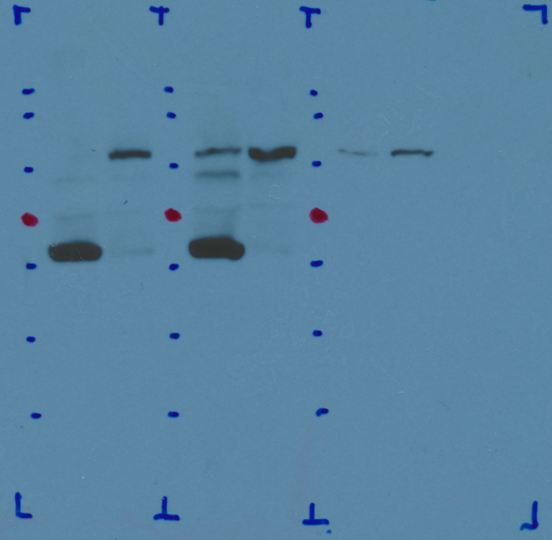

Supplement: Source data 1. [file elife-75143-data1.zip › Fig. 4_Figure supplement 1B -short expo - Phospho Snf1_Raw.tif]

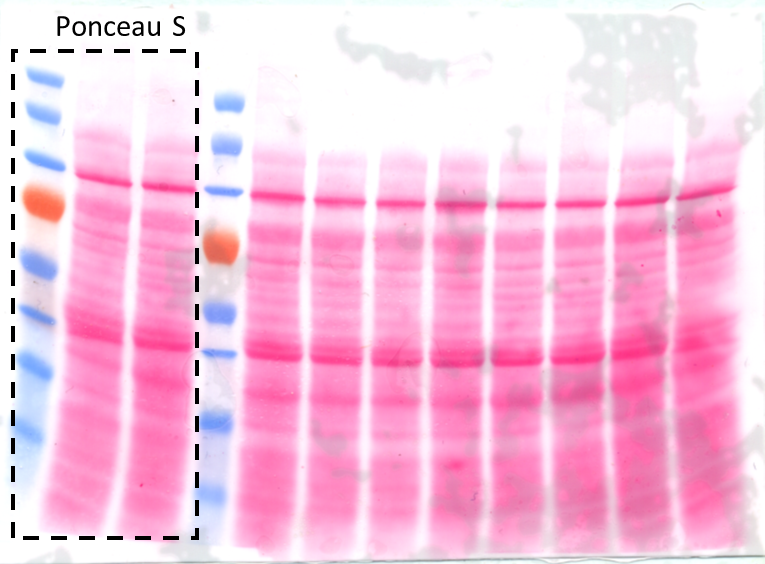

Supplement: Source data 1. [file elife-75143-data1.zip › Fig. 4A - Ponceau S_Annotated.tif]

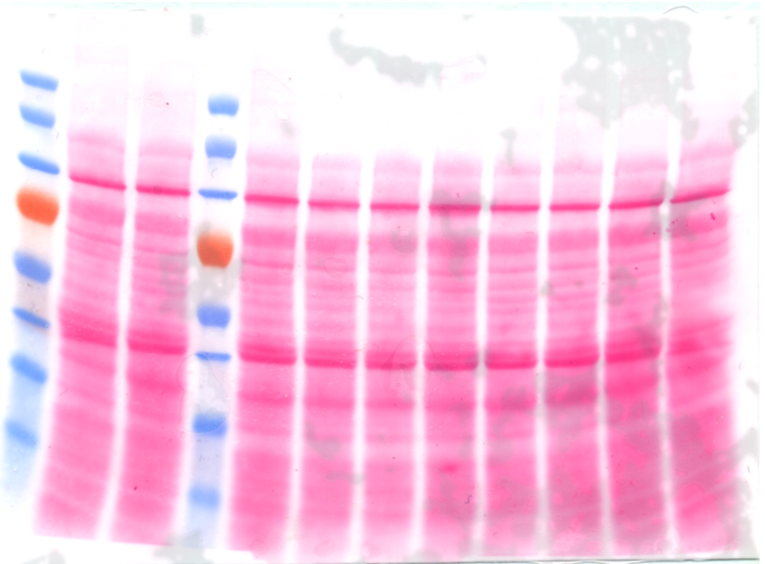

Supplement: Source data 1. [file elife-75143-data1.zip › Fig. 4A - Ponceau S_Raw.tif]

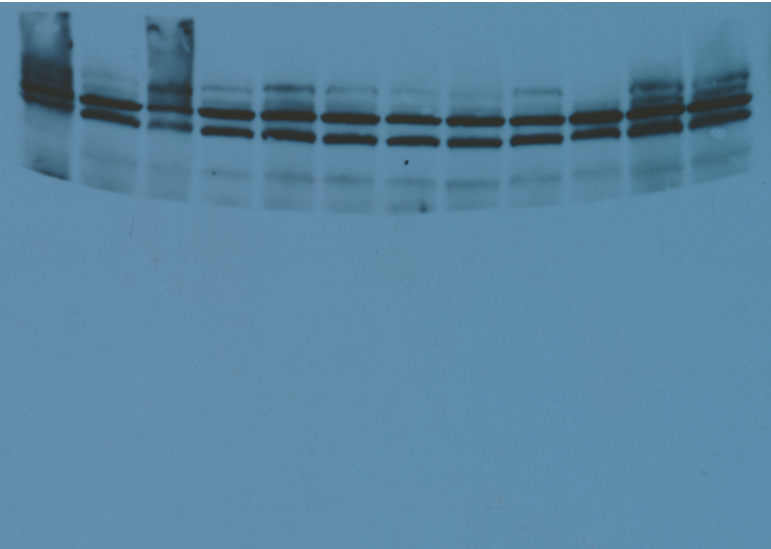

Supplement: Source data 1. [file elife-75143-data1.zip › Fig. 4C +glucose - Aox1_Raw.tif]
